# Plant-based enteral nutrition outperforms ultra-processed formulas in mitigating consequences of antibiotic-induced dysbiosis

**DOI:** 10.1172/jci.insight.199827

**Published:** 2026-04-09

**Authors:** Mona Chatrizeh, Jianmin Tian, Matthew Rogers, Firuz Feturi, Guojun Wu, Brian Firek, Roman Nikonov, Lauren Cass, Alexandra Sheppeck, Lavnish Ojha, Ali Carroll, Matthew Henkel, Justin Azar, Rajesh K. Aneja, Brian Campfield, Dennis Simon, Michael J. Morowitz

**Affiliations:** 1 Department of Surgery, University of Pittsburgh School of Medicine, Pittsburgh, Pennsylvania, USA.; 2 UPMC Children’s Hospital of Pittsburgh, Pittsburgh, Pennsylvania, USA.; 3 Division of Pediatric Infectious Diseases, Department of Pediatrics, University of Pittsburgh School of Medicine, Pittsburgh, Pennsylvania, USA.; 4 Department of Critical Care Medicine, University of Pittsburgh, Pittsburgh, Pennsylvania, USA.; 5 Division of Pediatric Critical Care Medicine, Safar Center for Resuscitation Research, UPMC Children’s Hospital of Pittsburgh, University of Pittsburgh, Pittsburgh, Pennsylvania, USA.

**Keywords:** Gastroenterology, Immunology, Microbiome

## Abstract

Malnutrition, gut inflammation, and antibiotic-induced dysbiosis are well-recognized risk factors for poor clinical outcomes among critically ill patients. We previously showed that commercially available plant-based enteral nutrition (PBEN) preserves a commensal microbiome compared with commonly used artificial enteral nutrition (AEN). In this study, PBEN was superior to AEN in promoting recovery from antibiotic-induced dysbiosis in mice and humans. PBEN effectively mitigated anemia and leukopenia, restored naive lymphocyte populations, and reduced bone marrow myeloid expansion. Animals randomized to PBEN also exhibited improved responses to infectious gastrointestinal challenges following antibiotic exposure. A pilot clinical study validated these findings, demonstrating increased gut commensals, reduced pathogens, and improved leukocyte balance in critically ill children receiving PBEN compared with AEN. Together, these results suggest that PBEN offers a practical dietary approach to mitigate antibiotic-associated complications and potentially improve clinical outcomes among hospitalized patients requiring supplemental nutrition.

## Abstract 2

Plant-based tube feeding helps rebuild healthy gut bacteria and restore immune cells after antibiotics—offering a simple dietary fix for hospitalized patients.

## Introduction

Critically ill patients exhibit dysbiotic microbiomes due to various factors, including the prevalent use of antibiotics (ABX) (1). While antibiotic use in the intensive care unit (ICU) is understandable given the severity of illness, ABX treatment is associated with poor outcomes beyond the well-known risk of antimicrobial resistance (2). In fact, ABX are more likely than any other class of drugs to be associated with adverse drug events (ADEs), including gastrointestinal (GI), hematologic, and infectious complications, prolonged hospitalization, increased cost, and mortality (3, 4). Even in the absence of detectable ADEs, ABX treatment is associated with poor outcomes in specific clinical contexts such as readmission for sepsis within 90 days of hospital discharge and complications after elective surgery (5, 6). In addition, researchers have reported increased mortality and morbidity following ABX depletion of the microbiome in a range of animal models, including bacterial pneumonia, fungal sepsis, and experimental pancreatitis (7–9). The underlying mechanisms of these complications are not fully understood, but it is clear that eradicating the gut microbiome with ABX can be harmful.

Despite improved stewardship programs, antimicrobial usage during critical illness is likely to remain prevalent, underscoring the need for strategies to restore gut microbial community structure, metabolic capacity, and host-protective functions after ABX exposure. Current approaches such as probiotics and fecal microbiota transplantation are limited by safety concerns (10, 11). Dietary interventions are a less explored strategy for mitigating ICU dysbiosis, with emerging evidence suggesting that dietary composition can modify both antibiotic efficacy and the unintended consequences of antibiotic use (12–14).

The most common form of supplemental nutrition for patients unable to eat by mouth consists of commercially available liquid formulas containing prespecified amounts of macro- and micronutrients, which we refer to as conventional or artificial enteral nutrition (AEN). In this study, we compared Vital (Abbott Laboratories; AEN), a chemically defined formula containing isolated whey and soy proteins with minimal fiber (1 g/8 fl oz), added sugars (6 g/8 fl oz), and proinflammatory preservatives and emulsifiers (15–18), against Liquid Hope (Functional Formularies; plant-based enteral nutrition [PBEN]), an all-natural formula derived from organic fruits, vegetables, and grains providing 3.7-fold higher fiber content (3.7 g/8 fl oz) and without added sugars and artificial additives (full nutritional profiles in Supplemental Table 4; supplemental material available online with this article; https://doi.org/10.1172/jci.insight.199827DS1). We and others have shown that PBEN is well tolerated in humans and prevents gut inflammation in murine models by promoting the growth of commensal anaerobes (19, 20). However, the comparative efficacy of these formulas in mitigating antibiotic-induced dysbiosis has not been systematically evaluated.

This study compares how AEN and PBEN affect antibiotic-induced dysbiosis. Murine models evaluated how these diets impact gut microbiota composition, diversity, and inferred functional capacity, alongside immune cell flux and susceptibility to infection following broad-spectrum ABX exposure. Rather than using experimental rodent formulas to study individual dietary components such as fiber or glucose, we directly examined intact commercially available human formulas in our murine model as others have done (21–24). This approach enhances translation relevance by modeling real-world clinical nutrition, and because these formulas are already in clinical use, our findings have immediate clinical applicability. Additionally, we conducted a single-center trial involving critically ill children randomized to either AEN or PBEN to assess the clinical implications of enteral formula choice for the gut microbiome. The clinical findings closely mirrored the preclinical results, and we observed a consistent association between enteral diet and lymphopenia, a clinically validated predictor of survival (25, 26).

## Results

### PBEN mitigates antibiotic-induced dysbiosis in mice.

**Figure 1:**
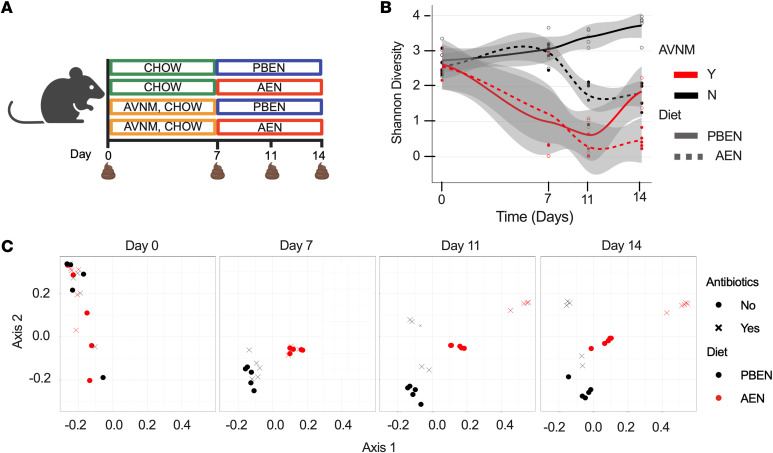
PBEN is superior to AEN in restoring microbial diversity following ABX exposure. (**A**) Experimental schematic. (**B**) α-Diversity (Shannon index) of stool microbiota from mice across dietary and antibiotic conditions (*n* = 4–5 per group). (**C**) Weighted UniFrac–based β-diversity showing clustering of microbial communities; each point represents 1 mouse. Samples with fewer than 1,000 reads were excluded from β-diversity analysis.

The composition and function of the microbiome are shaped by antibiotic use and diet. We used 16S rRNA gene sequencing to monitor temporal response to antibiotic and dietary interventions. Mice received a cocktail of broad-spectrum oral antibiotics (ABX; described in Methods) for 7 days, followed by a recovery period in which they were randomized to receive either plant-based enteral nutrition (PBEN) or AEN for an additional 7 days (Figure 1A). Sequencing data revealed a significant decline in α-diversity, measured by Chao1 and Shannon diversity indices, in all antibiotic-exposed animals indicating a reduction in microbial richness and diversity (Figure 1B, Supplemental Figure 1A, and Supplemental Tables 1 and 2). α-Diversity collapsed from baseline (Chao1 PBEN: 76.3 ± 5.6; AEN: 79.4 ± 7.0) after ABX treatment (PBEN: 14.4 ± 5.6; AEN: 18.7 ± 5.4). Importantly, PBEN-fed animals demonstrated significant recovery after 7 days of diet (Chao1: 33.4 ± 7.1) compared with AEN-fed animals (4.9 ± 0.6; *P* = 0.011) (Supplemental Figure 1A and Supplemental Table 2), suggesting superior microbiota restoration with plant-based nutrition. Importantly, AEN feeding alone also significantly reduced α-diversity even in the absence of ABX, with day 14 Chao1 richness substantially lower in AEN-fed mice (53.6 ± 10.0) compared with PBEN (125.4 ± 20.9; *P* = 0.008) (Supplemental Figure 1B and Supplemental Table 2). Two additional experimental iterations varied diets during ABX and recovery phases: all animals received AEN instead of normal chow during ABX (Supplemental Figure 1B), or half received PBEN and half received AEN during ABX treatment (Supplemental Figure 1C). In both cases, PBEN was consistently superior in maintaining α-diversity and mitigating dysbiosis compared with AEN, suggesting that PBEN supports microbiota richness whereas AEN leads to diversity loss both independently and after ABX perturbation.

β-Diversity analyses of community composition demonstrated similar baseline fecal microbiota across groups prior to dietary or antibiotic intervention (Figure 1C). Weighted UniFrac Principle Coordinates Analysis (PCoA) showed that PC1 and PC2 explained 16% and 10% of the variation, respectively. Permutational multivariate ANOVA (PERMANOVA) confirmed significant effects of time (*R*^2^ = 0.147, *P* = 0.001), antibiotic treatment (*R*^2^ = 0.085, *P* = 0.001), and diet (*R*^2^ = 0.037, *P* = 0.001) on microbial community composition (Supplemental Table 3). ABX-treated animals clustered separately from animals that did not receive ABX. During recovery, PBEN and AEN mice clustered distinctly from antibiotic-naive and antibiotic-treated groups (Figure 1C). Notably, the gut microbiota of PBEN-fed mice recovering from ABX showed greater similarity to that of ABX-naive animals, whereas AEN-fed mice remained persistently perturbed (Figure 1C). These results show that ABX alter gut microbial communities and diet shapes recovery.

**Figure 2:**
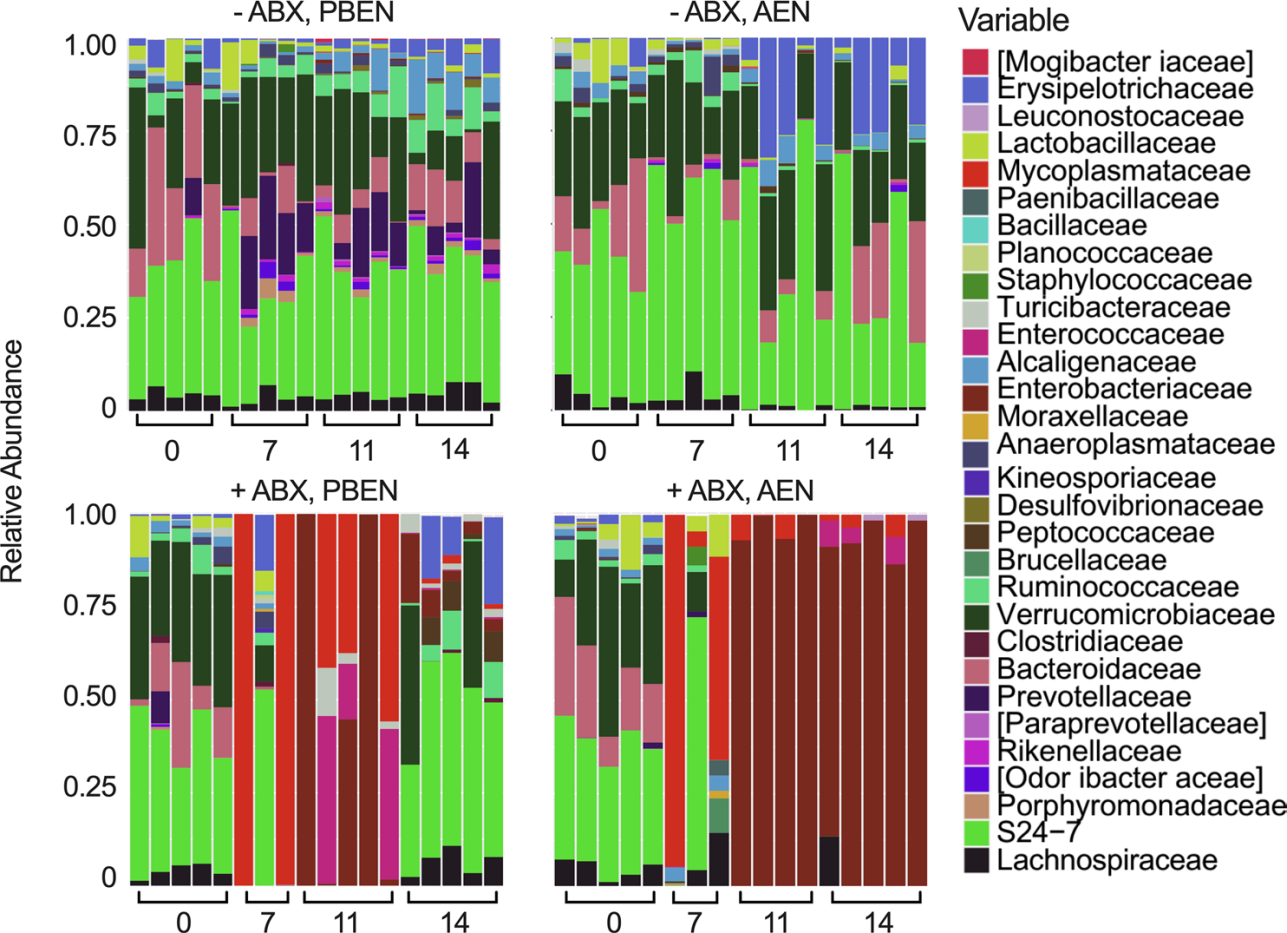
Diet shapes taxonomic composition following antibiotic exposure. Relative abundance of dominant bacterial families in fecal samples from mice receiving PBEN or AEN with or without antibiotic treatment (*n* = 4–5 per group). Each column represents an individual mouse.

We also observed diet-dependent taxonomic differences in gut microbial profiles (Figure 2). In the absence of ABX, PBEN-fed animals harbored more short-chain fatty acid–producing commensals, including Bacteroidetes, Ruminococcaceae, and Lachnospiraceae. AEN samples were enriched in taxa with pathogenic or mucus-degrading potential, such as Erysipelotrichaceae and Verrucomicrobiaceae, previously shown to bloom with fiber-deficient diets (27–30). After 1 week of ABX treatment, PBEN and AEN communities were both disrupted, but PBEN-randomized mice showed more favorable recovery with restoration of beneficial commensals, whereas AEN-randomized samples were almost uniformly dominated by Enterobacteriaceae (Figure 2). Confirmation experiments in an independent cohort also revealed pathogens in post-antibiotic AEN samples (Enterobacteriaceae and Enterococcaceae) (Supplemental Figure 2). Thus, diet critically determines post-ABX microbial recovery, with PBEN promoting reconstitution of beneficial commensals and AEN contributing to a pathogen-dominated dysbiotic state.

### AEN sustains GI inflammation during recovery from ABX treatment and promotes bacterial dissemination.

**Figure 3:**
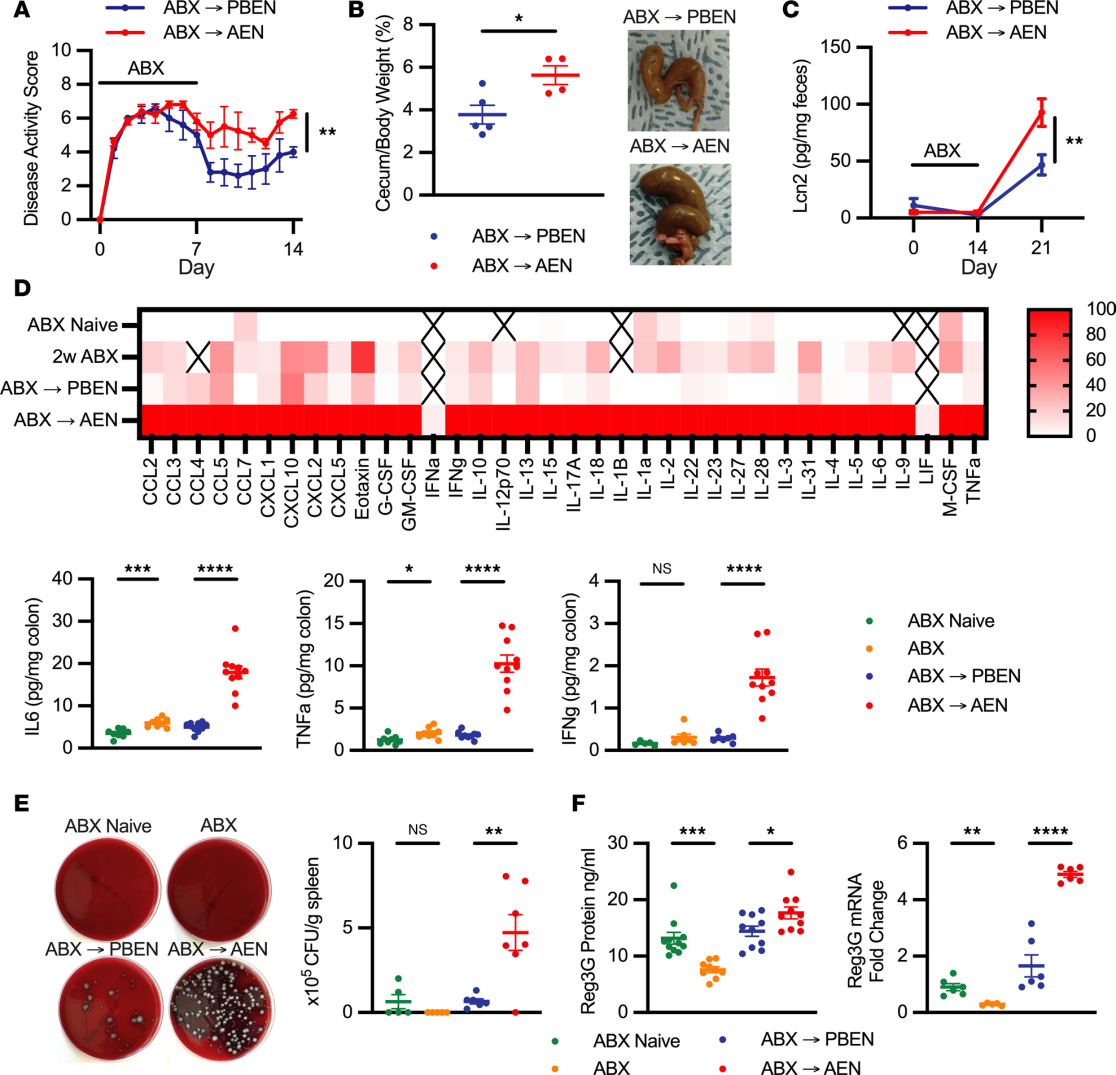
PBEN but not AEN mitigates signs of intestinal inflammation due to ABX exposure. (**A**) Disease activity index (DAI) following antibiotic treatment and dietary intervention (*n* = 5 per group). (**B**) Cecal/body weight ratio in PBEN (*n* = 5) versus AEN (*n* = 4) mice with representative cecal images. (**C**) Fecal *Lcn2* levels (*n* = 11 per group). (**D**) Heatmap of relative expression of 32 cytokines from colon tissue lysates (*n* = 6–11 per group); X denotes undetectable cytokines. Selected cytokines (IL-6, TNF-α, IFN-γ) are shown as pg/mg protein. (**E**) Splenic bacterial burden (*n* = 5–7 per group) with representative images. (**F**) Ileal *Reg3g* protein and mRNA expression (*n* = 5–11 per group). Data represent mean ± SEM. Significance: 2-tailed Student’s *t* test comparing ABX-naive vs. ABX-treated and PBEN vs. AEN after ABX. *P < 0.05, **P < 0.01, ***P < 0.001, ****P < 0.0001.

GI distress is a well-documented consequence of antibiotic treatment in rodents and humans (3, 31). In our experiments, antibiotic-treated animals exhibited intestinal distress similar to that seen in colitis models. Intestinal inflammation severity was assessed using a modified disease activity index (DAI) scoring weight loss, stool consistency, and hematochezia. Regardless of diet, animals exposed to ABX uniformly had increased DAI scores (Figure 3A). Mice receiving PBEN during washout experienced an improvement in symptoms, whereas disease severity persisted in AEN-fed animals (DAI PBEN: 4.00 ± 0.32; AEN: 6.25 ± 0.25; *P* = 0.0011). Building on prior observations that ABX increase cecal size and weight (31), we demonstrate that this response is diet dependent. AEN animals showed greater cecal/body weight ratios (5.6% ± 0.4%) than PBEN-fed animals (3.8% ± 0.4%; *P* = 0.022) (Figure 3B). As also reported by others, histologic examination of colonic tissue revealed no overt intestinal damage after ABX (not shown). However, enlarged submucosal lymphoid aggregates were observed after gut recolonization, particularly in AEN-fed mice (Supplemental Figure 3A) (32). Such lymphoid structures are associated with inflammatory bowel disease and suggest a gut-specific immune response (33–35). Finally, we measured fecal lipocalin-2 (*Lcn2*) as a biomarker of intestinal inflammation (36). Previous studies have shown that ABX treatment decreases LCN2 levels (37, 38). We extend these observations by demonstrating that during recolonization, LCN2 levels are diet dependent, with AEN-fed animals exhibiting higher LCN2 (day 14: 92.8 ± 12.0 pg/mg stool) than PBEN-fed (46.7 ± 8.9 pg/mg stool; *P* = 0.0062) (Figure 3C). This pattern suggests that post-antibiotic intestinal inflammation is influenced by diet and is more pronounced with AEN.

To assess ABX-induced intestinal inflammation and diet-dependent recovery, we performed multiplex Luminex analysis quantifying protein expression of 32 cytokines in colon tissue lysates. In some cases, e.g., IL-6 and TNF-α, we observed modest increases in proinflammatory cytokines immediately after completion of ABX. During recovery, AEN-fed mice exhibited global cytokine surge across all inflammatory and regulatory pathways, including G-CSF, GM-CSF, IFN-γ, TNF-α, IL-10, and IL-6, consistent with a dysregulated post-antibiotic immune response (Figure 3D). Interestingly, leukemia inhibitory factor (LIF), an IL-6 superfamily cytokine secreted by inflamed gut epithelium, was undetectable in all samples except AEN samples collected during the washout period (Figure 3D) (39, 40). To test whether microbiome reestablishment contributes to the post-antibiotic inflammatory response, we developed a coculture system in which immortalized HT29 epithelial cells were incubated with fecal samples from antibiotic-treated mice during PBEN or AEN washout and IL-8 expression, a mediator of GI inflammatory response, was analyzed (41–43). After 1 week of recovery, both diets induced IL-8 expression, suggesting that recolonization is proinflammatory. IL-8 expression was higher with AEN samples than PBEN, although this did not reach statistical significance. Coculturing HT29 with heat-killed bacteria from the same stool reduced this difference (Supplemental Figure 3B), suggesting that viable bacteria mediate the observed diet-dependent effects.

Gut inflammation and antibiotic-induced microbiota depletion both compromise intestinal barrier integrity, facilitating translocation of bacteria and their by-products (44–50). We therefore quantified bacterial colony-forming units (CFU) recovered within the spleens of antibiotic-treated animals before and after dietary randomization. As expected, only minimal bacterial CFU were recovered from spleens of ABX-naive or ABX-treated animals. However, we observed substantial bacterial growth in spleens collected 1 week after ABX cessation. AEN-fed animals during washout exhibited heavy splenic bacterial burdens (4.73 × 10^5^ ± 1.06 × 10^5^ CFU/g), exceeding those seen in PBEN-fed animals (0.663 × 10^5^ ± 0.151 × 10^5^ CFU/g; *P* = 0.0048) (Figure 3E). These data confirm prior reports that gut bacterial translocation peaks after ABX cessation and indicate that diet modulates its magnitude (46, 50). Given the known role of *Muc2* and *Reg3g* as regulators of mucosal immunity and intestinal inflammation, we hypothesized that their expression would be altered by ABX-induced microbiome changes. In line with observations by other groups that ABX disrupt the colonic mucus barrier, we found that ABX decreased colonic MUC2 expression, with no diet-dependent differences during recovery (Supplemental Figure 3C) (51, 52). Similarly, we confirmed previous findings that ABX treatment decreases ileal REG3G expression (Figure 3F) (53). As with fecal lipocalin, the increase in REG3G was more substantial with AEN (17.7 ± 1.0 ng/mL) during the washout period compared with PBEN (14.4 ± 0.9 ng/mL; *P* = 0.0314), likely reflecting microbial blooms during recolonization given the bactericidal activity of *Reg3*.

### AEN sustains an expansion of myeloid cells and contraction of lymphoid cells in the bone marrow after ABX.

**Figure 4:**
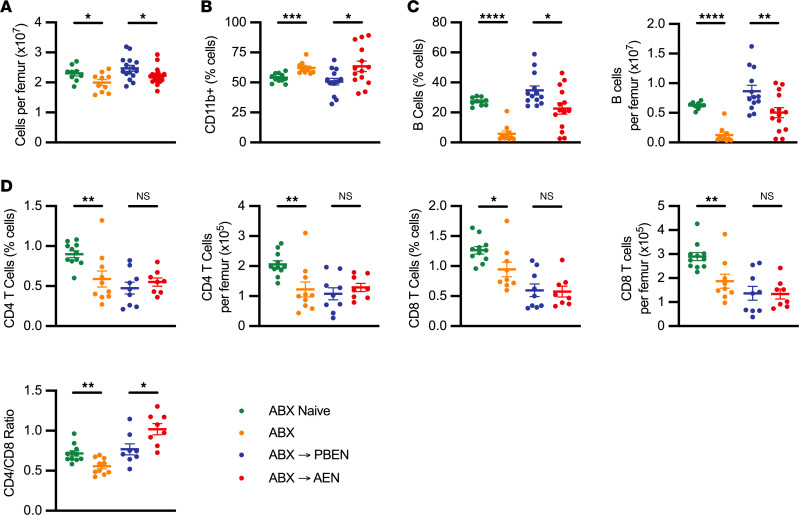
AEN sustains bone marrow myeloid expansion after ABX. (**A**) Bone marrow cellularity. (**B**) CD11b^+^ myeloid cell frequency. (**C**) B cell frequency and count. (**D**) CD4^+^ and CD8^+^ T cell frequencies, counts, and CD4/CD8 ratio (*n* = 7–14). Data represent mean ± SEM. Significance: 2-tailed Student’s *t* test comparing ABX-naive vs. ABX-treated and PBEN vs. AEN after ABX. *P < 0.05, **P < 0.01, ***P < 0.001, ****P < 0.0001.

Previous studies demonstrate that broad-spectrum ABX disrupt steady-state hematopoiesis by impairing basal *Stat1* signaling in the bone marrow niche, leading to reduced cellularity and skewing lineage development (54). Consistent with these findings, we observed a reduction in total bone marrow cellularity following ABX exposure (Figure 4A). In the absence of ABX, PBEN and AEN diets alone did not significantly differ in bone marrow cellularity (Supplemental Figure 4A). During ABX recovery, however, PBEN-fed mice exhibited significantly higher bone marrow cellularity (2.47 × 10^7^ ± 0.10 × 10^7^ cells) compared with AEN-fed (2.22 × 10^7^ ± 0.06 × 10^7^; *P* = 0.0333) (Figure 4A). CD11b staining of bone marrow cells by flow cytometry revealed increased myeloid cell frequency immediately following ABX treatment. Importantly, PBEN more effectively mitigated this increase during recovery compared with AEN (PBEN: 50.7% ± 2.7%; AEN: 63.5% ± 4.3%; *P* = 0.020) (Figure 4B). To assess lymphoid populations, we broadly gated on CD11b^–^ cells and evaluated B220, CD4, and CD8 expression, consistent with the approach used by Josefsdottir et al. (54). This gating strategy replicated published methods, enabling direct comparison with established ABX-induced hematopoietic changes. Using this approach, we observed that ABX reduced B cell frequency (CD11b^–^B220^+^) and the CD4/CD8 (CD11b^–^CD4^+^/CD8^+^) ratio in bone marrow cells, as previously reported (Figure 4, C and D) (54). During recovery, PBEN mice exhibited improved B cell restoration (34.7% ± 3.0%) compared with AEN (22.6% ± 3.7%; *P* = 0.020) (Figure 4C). The CD4/CD8 ratio was normalized in PBEN-fed mice (0.77 ± 0.07) but increased significantly in the AEN group (1.02 ± 0.07; *P* = 0.023) (Figure 4D). Together, these findings propose that post-ABX bone marrow composition is diet dependent, with PBEN partially restoring hematopoietic balance and AEN exacerbating ABX-induced dysregulation.

### PBEN is superior to AEN in rescuing ABX-induced anemia and leukopenia.

**Figure 5:**
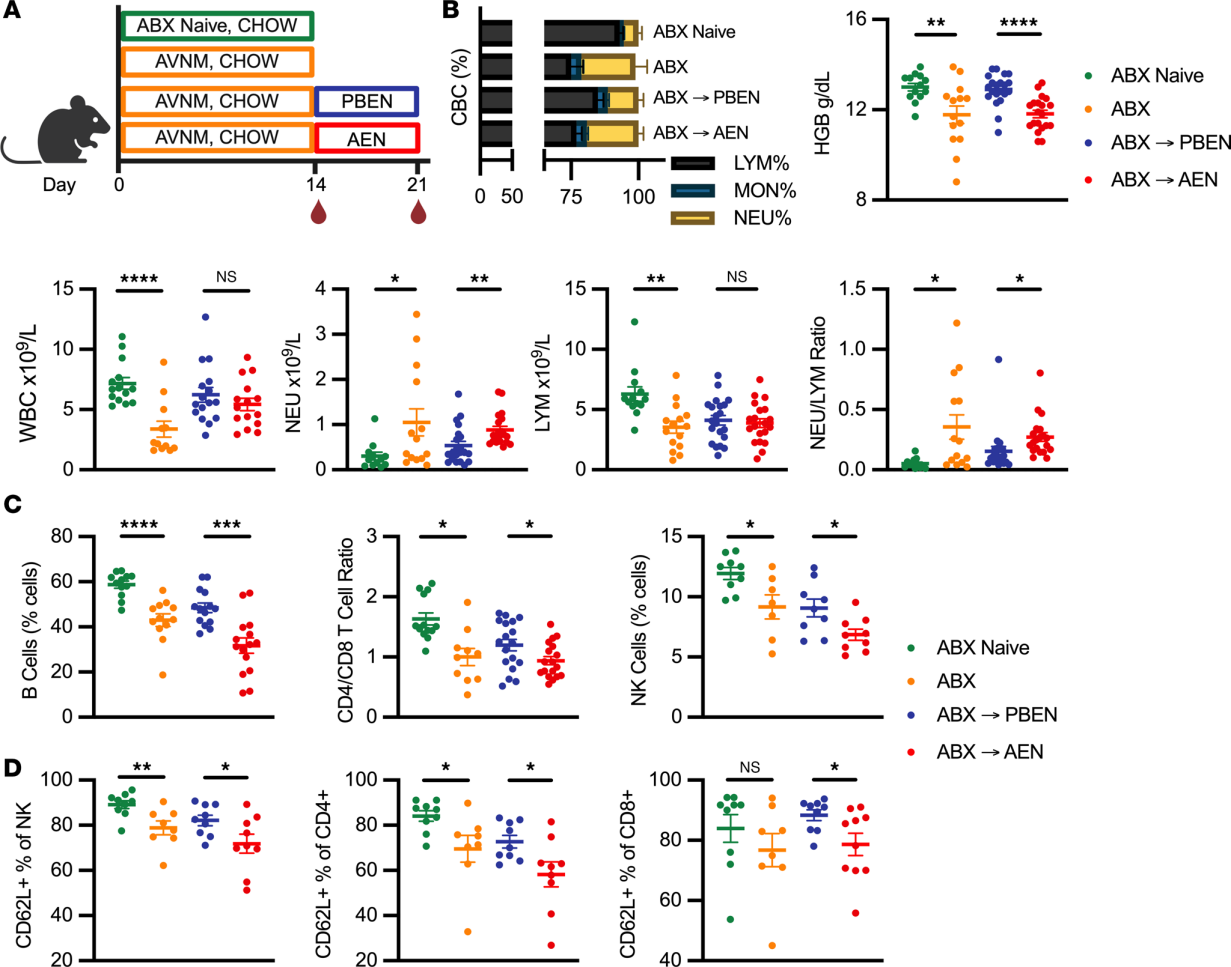
PBEN is superior to AEN in rescuing ABX-induced anemia and leukopenia. (**A**) Experimental schematic. (**B**) Peripheral blood profiles including differential, hemoglobin, total WBC, neutrophil (NEU), and lymphocyte (LYM) counts, and neutrophil-to-lymphocyte ratio. (**C**) Flow cytometric analysis of lymphocytes showing B cells, CD4/CD8 ratio, and natural killer (NK) cells with CD62L^+^ naive lymphocyte proportions (*n* = 8–15 per group). Data represent mean ± SEM. Significance: 2-tailed Student’s *t* test comparing ABX-naive vs. ABX-treated and PBEN vs. AEN after ABX. *P < 0.05, **P < 0.01, ***P < 0.001, ****P < 0.0001.

Clinicians have long recognized that some patients develop cytopenias during antibiotic treatment, but underlying mechanisms remained unclear until recent studies linked the microbiome and hematopoiesis (31, 54–57). Therefore, we assessed whether bone marrow immune cell shifts were reflected in peripheral blood counts (Figure 5A). In the absence of ABX, PBEN- and AEN-fed animals had similar complete blood count (CBC) profiles (Supplemental Figure 4B). As reported previously, antibiotic-induced dysbiosis led to anemia and leukopenia (Figure 5B) (54). However, mice fed PBEN during the recovery phase exhibited improved anemia, evidenced by higher hemoglobin levels (12.9 ± 0.1 g/dL) than in AEN-fed mice (11.8 ± 0.2 g/dL; *P* < 0.0001) (Figure 5B). ABX treatment also resulted in neutrophilia and leukopenia, resulting in a higher neutrophil-to-lymphocyte ratio (NLR), a marker of increased ICU mortality (58–61). While lymphocyte counts during washout were comparable between PBEN- and AEN-fed mice, AEN mice exhibited higher neutrophil counts and elevated NLR (AEN: 0.27 ± 0.04; PBEN: 0.15 ± 0.04; *P* = 0.036) (Figure 5B).

As in bone marrow analyses, we used flow cytometry to assess peripheral blood lymphocyte subsets within the CD11b^–^ fraction and found that ABX treatment decreased B (CD11b^–^B220^+^) and natural killer (NK; CD11b^–^NK1.1^+^) cells. As previously reported, CD4 (CD11b^–^CD4^+^) T cells decreased while CD8 (CD11b^–^CD8a^+^) T cells increased, resulting in a lower CD4/CD8 ratio (Figure 5C) (54). During recovery from ABX, PBEN mitigated most antibiotic-induced derangements, restoring lymphoid cells with higher B (PBEN: 48.4% ± 2.2%; AEN: 31.7% ± 3.4%; *P* = 0.0003), NK (PBEN: 9.1% ± 0.7%; AEN: 6.9% ± 0.5%; *P* = 0.0209), and CD4/CD8 ratios (PBEN: 1.20 ± 0.10; AEN: 0.94 ± 0.07; *P* = 0.0351) than AEN. We further investigated the activation state of these lymphocytes using CD62L (L-selectin), a marker of naive lymphocytes shed during homing to inflamed tissues (62–64). ABX treatment decreased the proportion of naive NK, CD4^+^, and CD8^+^ T cells, indicated by reduced CD62L^+^ population (Figure 5D). During recovery, PBEN was superior in restoring these naive populations in comparison with AEN-fed mice, which had lower proportions of naive NK, CD4^+^, and CD8^+^ T cells (Figure 5D). Collectively, these findings show that ABX disrupt peripheral immunity, favoring granulocytes over lymphocytes and altering naive-effector balance. PBEN was more effective than AEN in mitigating these effects and promoting hematologic recovery.

### PBEN minimizes severity of post-antibiotic vancomycin-resistant Enterococcus faecalis and Klebsiella pneumoniae GI infections but not Klebsiella-induced pneumonia.

**Figure 6:**
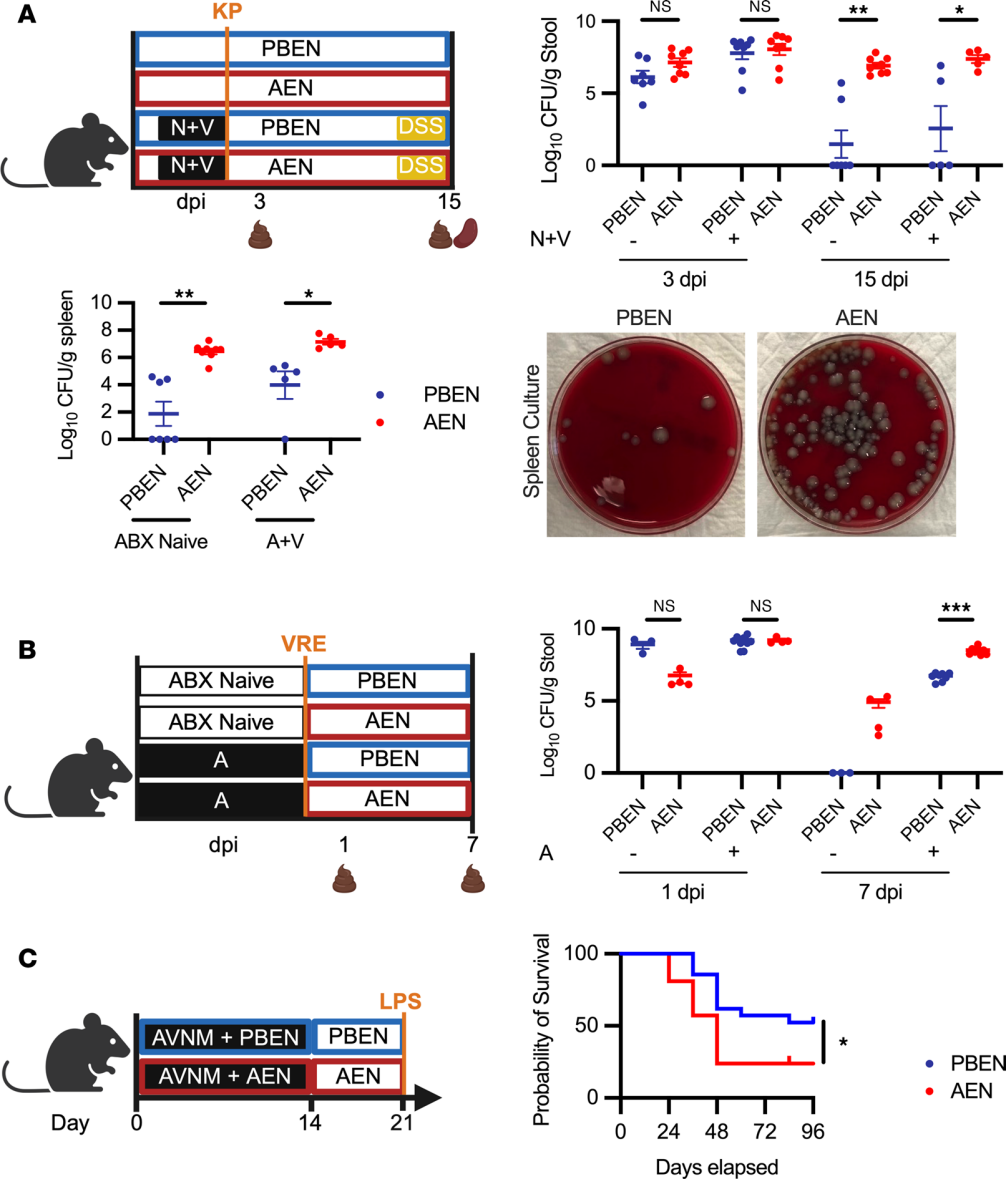
PBEN minimizes severity of post-ABX GI infections. (**A**) *K.* *pneumoniae*–DSS model schematic; *K. pneumoniae* 396 (KP) burden was quantified by qPCR in stool at 3 and 5 days post-infection (dpi) (*n* = 5–8 per group) and spleen at sacrifice (*n* = 5 per group). Representative spleen culture images demonstrate dissemination. (**B**) Vancomycin-resistant *Enterococcus faecalis* (VRE) model schematic; cecal VRE burden was assessed at 1 and 7 dpi (*n* = 4–9 per group). (**C**) Lipopolysaccharide (LPS) challenge schematic; Kaplan-Meier comparison of survival of PBEN- versus AEN-fed mice (*n* = 21 mice per diet), analyzed using the log-rank (Mantel-Cox) test. All other data represent mean ± SEM. Significance: 2-tailed Student’s *t* test comparing PBEN vs. AEN. *P < 0.05, **P < 0.01, ***P < 0.001.

We used murine models to test whether antibiotic- and diet-induced changes in peripheral blood and bone marrow influence susceptibility to infectious and inflammatory challenges relevant to humans receiving ABX and enteral nutrition. Using an established model of antibiotic-induced dysbiosis and gut barrier disruption, we tested PBEN and AEN effects on bacterial clearance and systemic translocation (65). PBEN- and AEN-fed mice were treated with neomycin and vancomycin, then inoculated with *Klebsiella pneumoniae* 396 (KP) by gastric gavage. Intestinal inflammation was induced 14 days post-infection (dpi) using dextran sulfate sodium (DSS) in ABX-treated animals. DSS was omitted in ABX-naive animals to isolate diet effects on an undisturbed microbiome. Fecal samples were collected to monitor KP colonization, and animals were sacrificed 3 days after DSS to assess splenic dissemination. Fecal KP burdens were similar in PBEN and AEN mice at 3 dpi. At 15 dpi, fecal KP burden and splenic translocation were significantly higher in AEN-fed than in PBEN-fed mice regardless of ABX exposure (Figure 6A). Interestingly, the protection offered by PBEN was not conferred in a KP-induced pneumonia. In this model, AEN-fed mice had more favorable outcomes than PBEN-fed (Supplemental Figure 6), suggesting that protection is limited to GI infections.

We next tested whether PBEN protection extends to another common nosocomial gut pathogen. Mice were randomized into 4 groups receiving either PBEN or AEN with or without ampicillin for 7 days. After ABX cessation, animals continued PBEN or AEN during a 7-day washout. After washout, all groups were gavaged with 10^5^ CFU of vancomycin-resistant *Enterococcus faecalis* (VRE) (66). While both ABX-naive PBEN and AEN groups had colonization and similar VRE burden at 1 dpi, PBEN-randomized animals had cleared the infection, whereas AEN mice had high VRE burdens (8.30 × 10^4^ ± 4.95 × 10^4^ CFU/g stool) at 7 dpi. With ABX pretreatment, PBEN-fed mice had significantly lower VRE burdens (5.49 × 10^6^ ± 1.00 × 10^6^ CFU/g stool) than AEN-randomized mice (3.39 × 10^8^ ± 0.78 × 10^8^ CFU/g stool) (Figure 6B). These results suggest that PBEN is superior to AEN in promoting clearance of intestinal VRE.

In a third evaluation of diet-dependent post-ABX inflammatory responses, mice received ABX for 2 weeks followed by 1 week of recovery on PBEN or AEN. On the final day of recovery, they received a potentially lethal dose of lipopolysaccharide (LPS) and were monitored for survival. Over the course of 3 separate cohorts, PBEN-fed mice demonstrated reproducibly increased survival compared with AEN-fed (Figure 6C). PBEN resulted in 37.5% mortality with survivors remaining healthy, while AEN resulted in 62.5% mortality with a median survival time of only 48 hours, illustrating that PBEN offers a protective effect against LPS-induced lethality. Overall, these data suggest that PBEN after antibiotic treatment offers a distinct advantage in inflammatory recovery and bacterial clearance in mouse models.

### Dietary intervention with pediatric PBEN promotes microbiome recovery and improved peripheral blood profile in critically ill children.

We previously demonstrated severe ICU dysbiosis in critically ill patients and separately showed that open-label conversion from AEN to PBEN in pediatric outpatients promotes commensal anaerobes and reduces gut pathogen colonization (19, 20, 67–69). Here, we conducted a small pilot study of children admitted to a single quaternary-care pediatric intensive care unit (PICU). All subjects were initiated on enteral feeds via feeding tube as part of routine clinical care, with no other changes to management. After consent, children were randomized 1:1 to commercially available pediatric PBEN (Nourish) or AEN (PediaSure) with the primary goal of comparing temporal changes in the gut microbiome between groups.

Thirty-two patients were enrolled, with 14 randomized to PediaSure and 18 to Nourish. Of these, 28 completed the study (13 PediaSure, 15 Nourish). Timing of enteral feed initiation was determined by clinicians without input from the research study team. Baseline characteristics, including race, sex, body mass index, comorbidities, and Pediatric Index of Mortality 3 (PIM-3) score, were similar (Table 1). Children randomized to PediaSure were younger (3.1 ± 2.3 vs. 6.1 ± 4.2 years; *P* = 0.03). There were no group differences in antibiotic exposure, positive cultures, or parenteral nutrition use. PICU and hospital length of stay (LOS) differed significantly between groups, with children receiving PBEN exhibiting longer LOS than those receiving AEN (Table 1). However, LOS in this small pilot study was highly variable and was influenced by a small number of patients with prolonged, complex hospital courses unrelated to diet. Importantly, all subjects received clinician-prescribed ABX, with no differences in exposure between PBEN and AEN groups.

**Figure 7:**
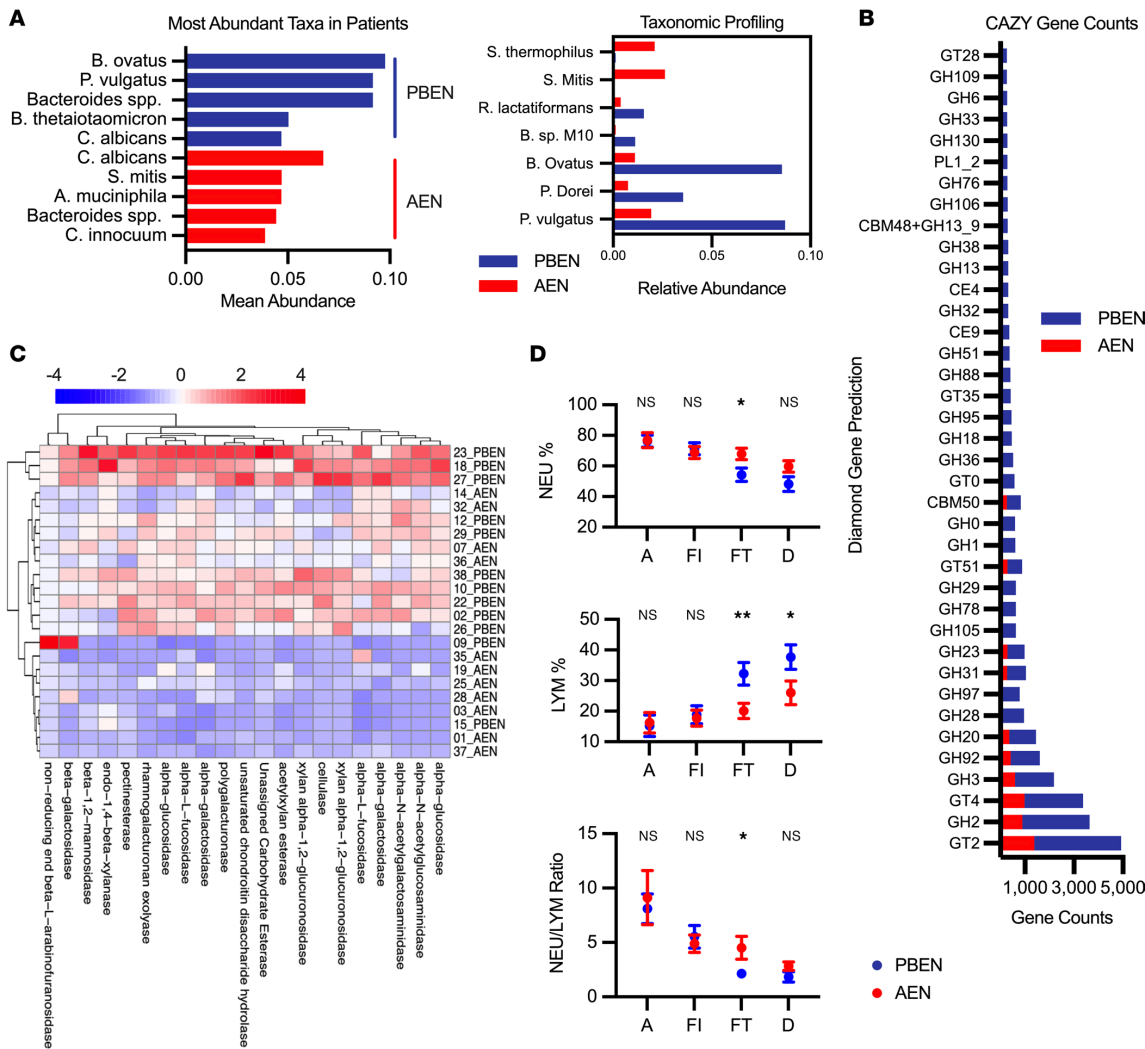
PBEN promotes microbiome recovery and improved blood profiles in critically ill children. (**A**) Most abundant taxa and relative abundance of microbes in PBEN versus AEN patients. (**B**) CAZy genes enriched in both diets (gene counts >250). (**C**) Polygalacturonase and β-glucosidase enrichment in PBEN versus AEN samples. (**D**) Neutrophil (NEU) and lymphocyte (LYM) percentages and NEU/LYM ratio at admission, feed initiation/termination, and discharge. Data represent mean and mean ± SEM. Statistical comparisons of microbial community composition were performed using PERMANOVA on Bray-Curtis distances. Complete blood count values were compared using unpaired Student’s *t* tests. *P < 0.05, **P < 0.01.

Metagenomic sequencing of fecal samples and rectal swabs confirmed similar baseline microbial diversity across groups. By contrast, at the conclusion of feeding, microbiome profiles differed markedly between groups (Supplemental Figure 7, A and B). Although pathogens such as *Candida albicans* remained detectable in both groups, the most abundant in PBEN samples included *Bacteroides ovatus* and *Phocaeicola vulgatus* (previously *B.* *vulgatus*), commensal anaerobes that degrade plant fiber to generate metabolites such as *N*-methylserotonin and short-chain fatty acids associated with protective intestinal inflammatory responses (70–72). In contrast, the most abundant taxa in AEN samples were the potential pathogens *Candida albicans* and *Streptococcus* (Figure 7A). Short-chain fatty acid–producing Bacteroidetes including *Bacteroides ovatus*, *Phocaeicola vulgatus*, and *Phocaeicola dorei* were far less prevalent in AEN fecal samples (Figure 7A). To characterize microbial functional capacity, we used the Carbohydrate-Active enZYmes database (CAZy) to identify diet-dependent carbohydrate metabolism enzymes. Overall, CAZy genes were highly upregulated (i.e., higher total gene counts) in PBEN patients compared with AEN (Figure 7B). Enriched CAZy genes in PBEN samples included polygalacturonase, a pectin-degrading enzyme, and β-glucosidase, which converts cellobiose to glucose during fiber hydrolysis (Figure 7C) (73, 74). These findings demonstrate that PBEN promotes microbiome recovery, reduces potential pathogens, and enhances beneficial commensals in critically ill children.

Although microbiome changes were the primary focus, we also analyzed CBC values in the pediatric cohort. These tests were ordered as part of routine care, and values were retrospectively extracted from medical records after study completion. There were no differences in WBC count, platelet count, or C-reactive protein at admission (Supplemental Figure 7C). There were also no differences in lymphocytes or neutrophils at admission or study diet initiation between groups. However, PBEN patients had a higher percentage of lymphocytes (PBEN: 32.23% ± 3.68%; AEN: 20.08% ± 2.48%; *P* = 0.0132) and a lower percentage of neutrophils (PBEN: 54.31% ± 4.39%; AEN: 67.92% ± 3.65%; *P* = 0.0270) among WBCs after the 2-week dietary intervention at feed termination (Figure 7D). Collectively, these results suggest that diet can influence microbiome composition and peripheral blood immune populations in critically ill patients.

## Discussion

Nutritional supplementation for hospitalized patients unable to eat by mouth remains controversial, with uncertainty surrounding the optimal timing, route, and quality of feeds. Although chemically defined enteral formulas have replaced pureed foods owing to ease of standardization, issues such as ingredient quality and biological impact have been largely overlooked. Few studies have examined how formula composition influences the microbiome or clinical outcomes. Whole-food alternatives offer higher fiber and fewer additives, potentially reducing dysbiosis and inflammation linked to added sugars, artificial sweeteners, and chemical emulsifiers that are present in AEN (15–18, 75). Additionally, fiber-enriched formulas may provide substrates that support beneficial short-chain fatty acid–producing bacteria and promote a healthier gut microbiome. Despite these advantages and known microbiome-independent benefits of fiber, the clinical impact of plant-based enteral formulas remains poorly studied (76).

This study examined how two commercially available enteral formulas, PBEN and AEN, modify the effects of ABX on the gut microbiome — an understudied but clinically important question given that many ICU patients receive both ABX and artificial nutrition. Using sequencing-based profiling, we found that gut microbial communities diverged significantly between PBEN- and AEN-fed animals even in the absence of ABX, with PBEN supporting higher overall diversity and greater abundance of commensal anaerobes such as Lachnospiraceae. These findings are consistent with prior human studies showing that low-fiber, highly processed enteral formulas are associated with reduced microbial diversity (77). Importantly, diet-dependent differences were amplified following antibiotic exposure, in line with prior work demonstrating that high-fat or fiber-deficient diets exacerbate antibiotic-induced dysbiosis, whereas fiber-rich diets promote microbial resilience (38, 78–82). The enhanced stability observed with PBEN may reflect its fermentable fibers and micronutrients, which provide a broader metabolic resource base than the low-fiber AEN formulation (Supplemental Table 4). However, these data do not allow us to distinguish whether PBEN is uniquely protective or AEN uniquely disruptive or whether the two diets influence microbial communities through distinct mechanisms.

Prior studies have shown that ABX disrupt gut barrier integrity and increase susceptibility to colitis, and broad-spectrum antibiotic regimens are commonly used to induce intestinal inflammation in murine models of inflammatory bowel disease (35, 49, 50). Consistent with this literature, we observed gross evidence of intestinal injury across all antibiotic-treated mice. However, we further found that both gut and systemic inflammation peaked during the post-antibiotic recolonization phase rather than during antibiotic exposure itself, as reflected by elevated fecal lipocalin-2, dysregulated colonic cytokines, and increased bacterial translocation after antibiotic cessation. This temporal pattern aligns with findings by Knoop et al., who reported bacterial translocation to mesenteric lymph nodes during the antibiotic washout period but not during active treatment (50). To our knowledge, aside from that study, few prior reports have specifically documented a delayed inflammatory surge during microbial recolonization following antibiotic cessation (50, 83). Importantly, we extend these observations by demonstrating that this post-antibiotic inflammatory response is strongly diet dependent, with significantly greater inflammation in AEN-fed animals compared with PBEN-fed animals.

Immune dysfunction during critical illness is strongly associated with poor outcomes, and prior work has shown that antibiotic-induced dysbiosis disrupts hematopoiesis — causing anemia, leukopenia, reduced bone marrow cellularity, and splenic atrophy through impaired gut microbe–bone marrow crosstalk (54–57, 84, 85). We recapitulated these abnormalities but further observed a paradoxical expansion of circulating myeloid cells and proinflammatory cytokines despite reduced lymphocytes and total leukocytes, revealing diet-modifiable myeloid skewing during ABX-associated inflammation. This phenotype is clinically relevant, as biomarkers of myeloid predominance, such as elevated neutrophil-to-lymphocyte ratios, are strongly linked to increased mortality in hospitalized patients (58–61). Importantly, we demonstrate that ABX-induced bone marrow dysfunction and myeloid skewing are diet dependent, with nearly all abnormalities exacerbated by AEN. Consistent with this immune rescue, PBEN conferred functional protection in murine models of infection and endotoxemia, improving survival, reducing bacterial dissemination, and enhancing pathogen clearance — highlighting nutrition as a modifiable determinant of immune recovery after antibiotic exposure.

Finally, we conducted a randomized nutritional intervention in critically ill children requiring enteral feeding, comparing commercially available pediatric PBEN and AEN formulas. Although antibiotic use was not protocolized, all enrolled participants received ABX during the study period. The trial was designed to assess diet-dependent changes in the gut microbiome rather than clinical endpoints such as mortality or nosocomial infection. Consistent with our murine findings, children receiving PBEN exhibited significantly lower neutrophil-to-lymphocyte ratios despite similar total leukocyte counts and C-reactive protein levels, suggesting mitigation of antibiotic-associated myeloid skewing.

Despite limited sample size, patient heterogeneity, and the confounding inherent to PICU cohorts, we detected clear diet-dependent differences in gut microbial diversity and taxonomic composition. PBEN samples were enriched in commensal anaerobes including *Bacteroides*, *Phocaeicola*, and *Ruthenibacterium*, whereas AEN samples were dominated by *Streptococcus* species such as *S.* *salivarius*. Interestingly, enrichment of oral microbes such as *S.* *salivarius* in the distal intestine has been identified as a biomarker of generalized depletion of the gut microbiome after antibiotic treatment (86–88). Although metabolomics were not performed, CAZy profiling revealed greater genomic capacity for dietary fiber degradation among PBEN-associated taxa, consistent with enhanced short-chain fatty acid production. Together, these findings are biologically plausible and align with prior work linking antibiotic exposure, added sugars, emulsifiers, and low-fiber diets to dysbiosis, while supporting a protective role for PBEN in microbiome and immune recovery in critically ill children.

Several limitations of this study warrant consideration. The animal model used does not fully reflect the complexity of the human ICU environment. While the liquid diets fed to mice differ considerably from standard rodent chow, we note that commercial enteral nutrition formulas used in the critical care setting are also distinct from typical human diets. Thus, while not physiologic, the dietary interventions in mice are directionally analogous to clinical practice. Additionally, the aggressive ABX regimen used in the study is routinely used in experimental models of antibiotic-induced dysbiosis. However, human patients often face similarly aggressive antibiotic exposures that are typically intravenous rather than enteral. Prior rodent studies of intravenous and intraperitoneal ABX as well as more limited oral ABX regimens have demonstrated similar patterns of antibiotic-induced dysbiosis and antibiotic-induced immune dysfunction, but future studies will need to determine how the route of ABX administration impacts these results. Additionally, our immune profiling was largely restricted to peripheral blood and bone marrow compartments, and lymphocyte subsets were identified by CD11b gating. Future work should employ comprehensive immunophenotyping panels to definitively characterize individual lymphoid and myeloid populations and analyze additional tissues, including the colon and associated mucosal immune compartments, as well as primary and secondary lymphoid organs. These assessments could reveal tissue-specific inflammatory signatures and further elucidate how nutritional interventions modulate local versus systemic immune responses following antibiotic-induced dysbiosis.

We acknowledge that the human pilot study was small in scale and patient characteristics, particularly age and length of stay (LOS), differed significantly between groups. Given that gut microbiota and mucosal immunity evolve rapidly in early life and resemble an adult-like state by approximately 3 years of age, age-related differences could influence both microbiome composition and immune tone (89–92). This disparity may confound the observed effects of diet on microbial communities and inflammatory markers. Additionally, children who received the PBEN intervention had longer ICU and hospital stays compared with those receiving AEN, which may reflect baseline illness severity or other confounding variables. Some children randomized to PBEN experienced unusually complex, prolonged hospital courses unrelated to nutrition assignment, including a patient who required more than 100 days of hospitalization, 2 newly tracheostomy/ventilator-dependent children, and a patient requiring multiple operative interventions and grafting after severe trauma. By contrast, several children in the PediaSure arm had short, uncomplicated admissions such as pneumonia or febrile seizure. These clinical outliers produced wide LOS distributions that are not fully captured by admission severity scores and underscore the inherent variability of pilot studies in critically ill populations. Therefore, while not necessarily indicative of harm with a specific enteral nutrition formula, the difference in LOS underscores the need for randomized, adequately powered studies to determine whether PBEN has a net clinical benefit in critically ill pediatric populations.

Critically ill patients experience rapid collapse of the gut microbiome, with more than 90% of commensals lost within 6 hours of critical illness and replaced by ultra-low-diversity communities dominated by a few pathobionts (93). Baseline microbial diversity appeared similar between groups at enrollment, and this widespread collapse in critically ill patients likely further reduces inter-individual variability. Still, residual differences in microbial resilience or metabolic capacity could influence the degree to which patients respond to nutritional interventions. Furthermore, our metagenomic and CAZy analyses suggest that PBEN enriches taxa with expanded carbohydrate-degrading capacity, but these findings remain correlative. Future work aimed at isolating contributing strains, mapping their metabolic outputs, and testing purified PBEN components will be essential to define the mechanisms through which diet shapes microbiome recovery and immune function following antibiotic exposure.

In summary, our findings identify enteral formula composition as a previously underappreciated determinant of microbiome recovery, immune reconstitution, and host resilience following antibiotic exposure. Across murine and PICU cohorts, plant-based enteral nutrition consistently supported greater microbial diversity, reduced post-antibiotic inflammatory rebound, and mitigated pathologic myeloid skewing — changes that translated into improved pathogen clearance and survival in experimental infection and inflammatory models. These data suggest that nutritional composition does not merely provide calories during critical illness but actively shapes the ecological and immunologic trajectory of recovery from antibiotic-induced dysbiosis. Although confirmation in larger, adequately powered clinical trials is required, our results raise the possibility that replacing low-fiber, ultra-processed formulas with whole food–based alternatives could represent a scalable and low-risk strategy to improve outcomes in critically ill patients, a population in which nearly all individuals experience profound microbiome disruption.

## Methods

Further information can be found in Supplemental Methods.

### Sex as a biological variable.

Male C57BL/6 mice were used, as prior studies demonstrate sex-specific differences in gut microbiome composition and immune responses. Both sexes were enrolled in the clinical trial; however, given the limited sample size, the study was not powered to assess sex-specific differences, and sex was not considered a variable.

### Mice.

Male C57BL/6 mice (8–10 weeks, 20–30 g; The Jackson Laboratory) were housed under standard 12-hour light/12-hour dark cycles. Mice were randomized to standard chow, PBEN, or AEN, with or without ABX (ABX: ampicillin 1 g/L, vancomycin 0.5 g/L, neomycin 1 g/L, and metronidazole 1 g/L, supplemented with Splenda [Heartland Food Products Group] 4 g/L) administered via drinking water or liquid diet. Liquid diets included the standard adult polymeric formula Vital (Abbott Laboratories; AEN) and Liquid Hope (Functional Formularies; PBEN), with nutritional composition detailed in Supplemental Table 4. To avoid social isolation effects on microbiome and immunity, mice were group-housed with cage-level feeding. PBEN was diluted 4:5 to match AEN caloric density. Each cage received 50 mL daily; complete cage-level consumption was verified. Individual mouse intake was not monitored, but body weights remained equivalent across groups (Supplemental Figure 8).

### Human samples.

**Figure 8:**
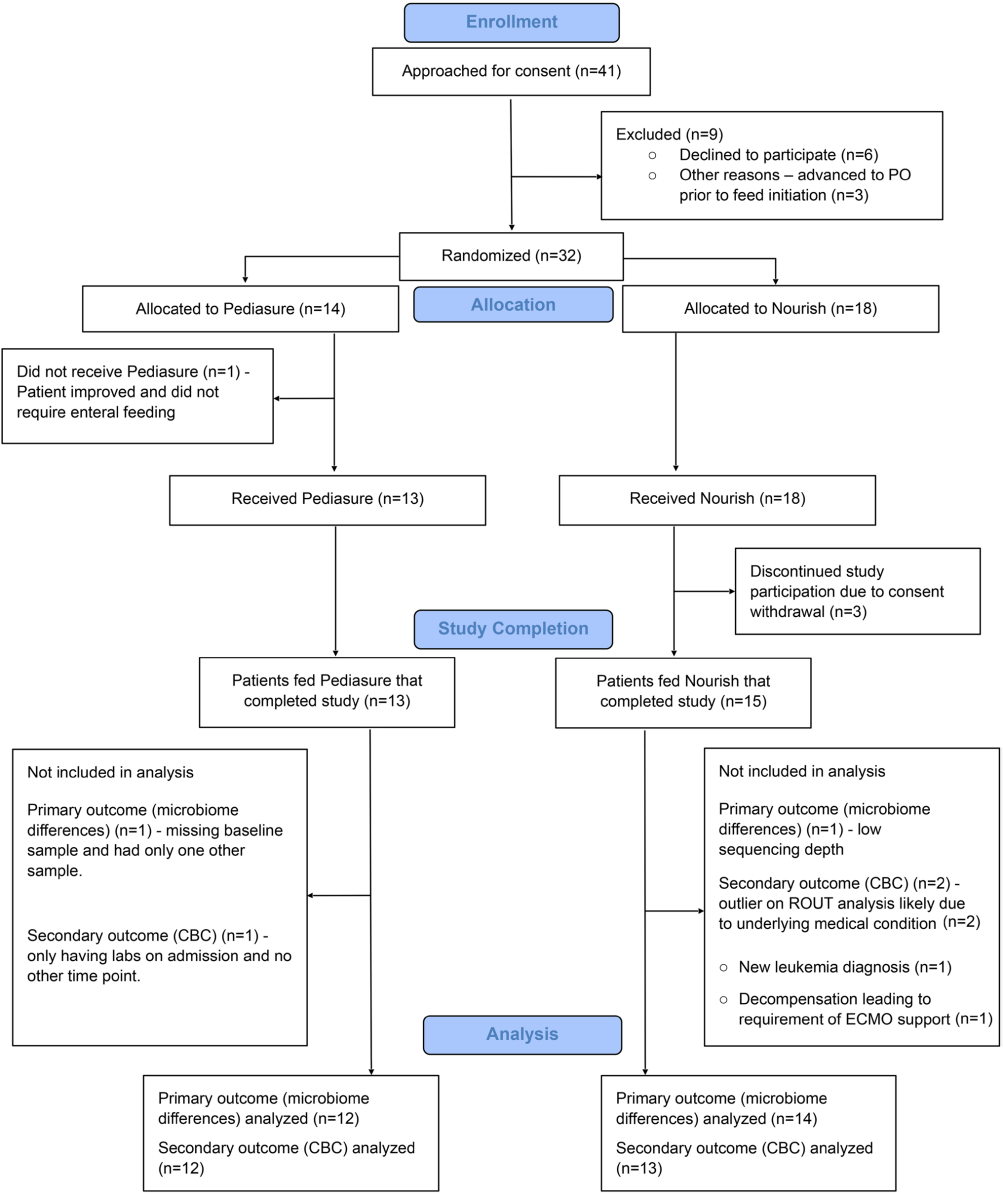
Modified CONSORT diagram. Flowchart of participants’ disposition throughout the study. ECMO, extracorporeal membrane oxygenation; PO, per os.

Thirty-two critically ill children admitted to the PICU at UPMC Children’s Hospital of Pittsburgh were randomized 1:1 to receive pediatric PBEN (Nourish, Functional Formularies) or AEN (PediaSure, Abbott Laboratories). Three patients withdrew, and 28 competed the study (13 AEN, 15 PBEN) (Figure 8). Nourish was diluted 4:1 with sterile water to prevent feeding tube obstruction. Decisions regarding enteral feeding (timing, route, and rate) were made by the treating clinical team. Nutritional consultation ensured adequate intake with both formulas. Exclusion criteria included vasopressor use; allergy or intolerance to either formula, whey protein, or gluten; and medical conditions requiring specialized nutrition (e.g., ketogenic diet for epilepsy).

Fecal samples and rectal swabs were collected at feed initiation and days 5–7 and 14 from patients remaining on enteral nutrition using BD BBL Culture Swabs and frozen at –80°C for batch processing as previously described (69). Demographic data, length of stay, positive cultures, study diet utilization, and CBC results were extracted from the medical records. Antibiotic coverage was scored from 0 to 4 based on coverage of anaerobic Gram-positive/negative and aerobic/anaerobic coverage. The study diet goal rate × hours was calculated as (actual volume of study diet administered / goal volume) × 24 to determine the amount of study diet received normalized to the goal rate set by the treating physicians.

Patients were enrolled by convenience sampling February 2018 to September 2019 with a target enrollment of 40 patients. Prior laboratory studies and clinical trials indicated that this sample size would provide adequate power to detect differences in gut microbial abundance between Nourish (PBEN) and PediaSure (AEN). A prior pilot study of enteral nutrition–dependent children transitioning from AEN to PBEN demonstrated that significant microbiome changes could be detected using baseline and post-intervention sampling, a design replicated here for the primary outcome (20). Secondary outcomes (CBC values, clinical metrics) were monitored but not powered for formal analysis. For secondary CBC analyses, 2 patients were excluded as outliers (ROUT and Grubbs’s tests): one with newly diagnosed leukemia and one with marked stress response to extracorporeal membrane oxygenation. No study-related harms occurred.

### 16S rRNA gene sequencing and analysis.

DNA extraction and 16S rRNA gene sequencing were performed at Microbiome Insights or Wright Labs. DNA was extracted using the QIAGEN PowerSoil kit. Amplicon PCR targeted the V3–V4 region of the 16S rRNA gene (515F/806R primers), followed by Illumina MiSeq sequencing (v2 chemistry, paired-end 250 bp reads). Sequences were processed using QIIME 2 (94). Amplicon sequence variants (ASVs) were generated with DADA2 (https://github.com/benjjneb/dada2) after trimming of 22 bp from read ends. Taxonomy was assigned using a naive Bayes classifier trained on the 515F/806R Greengenes database (v13.5) (95). A phylogenetic tree was generated using FastTree (96). ASV table, taxonomy, sequences, and trees were exported in hd5, tsv, fasta, and newick formats, respectively. Taxonomic assignments and metadata were combined with the ASV table into a single BIOM object using BIOM tools (https://biom-format.org/). This BIOM file along with the tree of ASVs was imported into R as a phyloseq object using the R phyloseq library (97).

Samples were rarefied for α- and β-diversity analyses using phyloseq and vegan R libraries (98). α-Diversity was measured using Chao1 and Shannon diversity metrics. Significance was tested using pairwise Wilcoxon’s tests. β-Diversity used weighted UniFrac and Bray-Curtis metrics, assessed with Adonis2 and PERMDISP algorithms in the vegan R library. Taxonomic abundances were compared using Analysis of Composition of Microbiomes 2 (ANCOM2) (99).

### Metagenomic sequencing.

DNA for metagenomic sequencing was extracted from stool and swab samples using a QIAGEN DNeasy 96 PowerSoil Pro QIAcube HT kit. Library preparation and sequencing were performed at Wright Labs on Illumina HiSeq (paired-end 150 bp reads). Reads were processed with cutadapt (primer removal) and Trimmomatic (quality trimming) and aligned against the human genome using Burrows-Wheeler Aligner (BWA); unmapped reads were returned (100–102). On average, 2.04 million reads per sample (standard deviation 1.9 million) were obtained. Taxonomic and functional profiles were generated using Kraken 2 with standard database (2022 build) (103). Kraken results were converted to a BIOM-format operational taxonomic unit (OTU) table using the kraken2biom script, and then the BIOM file was imported into phyloseq. Samples with fewer than 100,000 reads were removed from downstream analysis. α-Diversity was calculated using only the Shannon entropy metric, since, owing to the nature of shallow metagenomic data, OTU abundance tends to be overestimated. β-Diversity distances were calculated using only the Bray-Curtis metric, as UniFrac methods require a phylogenetic tree, which would require a universal marker between OTUs.

### Metagenomic assembly.

For each sample, an assembly of metagenomic reads were assembled with the MEGAHIT metagenomic assembler (104) using a range of k-mer sizes (21, 29, 39, 59, 79, 99, 119, and 141). Contigs from each assembly were assigned taxonomy using Kraken 2. To estimate the abundance of each contig, BWA was used to map the sample reads back to the contigs, and SAMtools was used to produce depth of reads per contig (105). Abundance per contig was normalized to library size and contig length in an equivalent sense to fragments per kilobase per million bases (FPKM) occasionally used in transcriptomic experiments.

### CAZyme annotation.

The CAZy gene–encoding contigs from the metadata were identified and classified based on the carbohydrate-active enzymes (CAZymes) database by the CAZymes Analysis Toolkit (CAT) (105). A combination of DIAMOND and HMMER predictions was used to annotate contigs encoding CAZymes (106, 107).

Putative plant cell wall polysaccharide-degrading enzymes belonging to different CAZy families were identified and classified based on sequence-based annotation. The CAZyme-encoding contigs were analyzed manually for different classes of CAZymes: glycoside hydrolases, glycoside transferases, carboxyesterases, carboxy-binding module family, and polysaccharide lyases. Subsequently, the CAZy results obtained were analyzed manually to determine the proportions of the different CAZymes present in the rumen metagenome data. Differential abundance testing of CAZymes was performed on reads per kilobase per million mapped reads (RPKM) values using multiple Wilcoxon’s tests. As many patients had only one sample, we selected one representative sample from each patient. Where multiple samples were present we chose samples that occurred multiple days after the diet started.

### Complete blood counts.

Peripheral blood was collected via cardiac puncture into EDTA tubes and analyzed using VetScan HM5 (Zoetis).

### Flow cytometry.

Mouse bone marrow was obtained by femur flushing. Blood and bone marrow cells were processed to remove red blood cells. Cell counts and viability were assessed using a Cellometer (Nexcelom). Surface staining was analyzed on a Cytek Aurora flow cytometer (Cytek Biosciences). After gating strategy for identification of populations (Supplemental Figure 5), data were analyzed using FlowJo.

### Lipocalin and REG3G ELISA.

Fecal samples (lipocalin) and ileal tissue (REG3G) were snap-frozen at –80°C. Samples were homogenized in PBS with protease/phosphatase inhibitors; supernatants were used for ELISA per the manufacturer’s instructions (Supplemental Key Resources Table). Assays were performed in duplicate; average values were used for data analysis.

### Luminex.

Colon tissue was snap-frozen in liquid nitrogen and stored at –80°C. Frozen samples were homogenized in RIPA buffer; protein concentrations were determined by bicinchoninic acid assay. Equal protein amounts were used for Luminex (Thermo Fisher Scientific) multiplex analysis on a MAGPIX instrument (Luminex Corporation).

### RNA extraction and qPCR.

RNA was isolated using QIAzol; concentration and integrity were assessed by NanoDrop (Thermo Fisher Scientific). Normalized RNA was transcribed to cDNA. qPCR was performed on QuantStudio 3 (Thermo Fisher Scientific) with SYBR Green Master Mix. Relative expression was calculated using ΔΔCt method normalized to GAPDH or β-actin.

### Spleen culture.

Spleens were aseptically collected, weighed, snap-frozen, and stored at –80°C. Thawed spleens were punctured and homogenized by vortexing with 1.4 mm ceramic beads in PBS (1 mL/g) for 10 minutes. Homogenates were serially diluted and plated on blood agar for 48 hours at 37°C. Colony counts were quantified per group.

### LPS inflammatory challenge.

Mice received ABX in PBEN or AEN for 2 weeks, followed by 1-week recovery on respective diets without ABX. Mice were then challenged intraperitoneally with LPS (*E. coli* O111:B4) at 10 mg/kg. Mortality was monitored twice daily for 1 week.

### Vancomycin-resistant Enterococcus infection.

An overnight culture of vancomycin-resistant *Enterococcus faecalis* strain 34-10-S (VRE; a gift from the Van Tyne Lab, University of Pittsburgh) was grown to saturation in BHI medium with 10 μg/mL vancomycin. Inocula of VRE were prepared for gavage by dilution to 5 × 10^6^ CFU/mL in sterile PBS. Mice were infected by gavage with 10^5^ CFU of VRE after being exposed to 7 days of ampicillin (1 g/L). Bacterial counts were determined by plating of serial dilutions of stool samples on agar plates with vancomycin. VRE colonies were identified by their ability to grow on vancomycin-treated bile-esculin agar plates.

### Klebsiella pneumoniae–colonized model of DSS-induced colitis.

Mice were randomized to receive PBEN or AEN for 3 days. Half in each group then received vancomycin (2.5 g/L) and neomycin (5 g/L) for 72 hours. On day 7, all mice were colonized with *Klebsiella pneumoniae* 396 (KP) via gavage (1 × 10^8^ CFU in 0.1 mL per mouse). Colitis was induced on day 19 with 4% dextran sulfate sodium (DSS) in drinking water. After 3 days of DSS treatment (day 22), mice were euthanized for tissue collection. Stool and spleens were collected throughout and at sacrifice. *K.* *pneumoniae* burden was quantified by qPCR using KP-specific primers (108).

### Statistics.

α-Diversity (Chao1 and Shannon indices) was compared using Mann-Whitney *U* tests for between-group comparisons and Wilcoxon’s signed-rank test for paired temporal comparisons. β-Diversity was assessed using weighted UniFrac (analyzed with Adonis2 and PERMDISP). Differential taxonomic abundance was assessed using ANCOM2. Murine experiments used unpaired 2-tailed Student’s *t* tests unless otherwise indicated. Survival was analyzed using log-rank (Mantel-Cox) tests. For the clinical study, microbial community composition was compared using PERMANOVA on Bray-Curtis distances; CBC values used Student’s *t* tests. Outliers were identified using ROUT and Grubbs’s analyses. *P* < 0.05 was considered significant.

### Study approval.

Mouse experiments were approved by the University of Pittsburgh IACUC (22040920) and Institutional Biosafety Committee (IBC201900106). The human study was approved by the University of Pittsburgh IRB (19080289) and registered at ClinicalTrials.gov (NCT03414775). Written informed consent was obtained from parents/legal guardians prior to enrollment.

### Data availability.

This study did not generate new unique reagents. Sequencing data have been deposited and are publicly available under BioProject PRJNA1200130. An Excel file titled Supporting Data Values, containing all supporting data for the main article and supplemental material, including raw data underlying all graphs, bar plots, scatterplots, and reported means, is provided. Additional information required to reproduce or reanalyze the findings is available upon request.

## Author contributions

MC, JT, and MJM conceptualized the study. MC, JT, MJM, and MH developed methodology. MR and JA used bioinformatics software. MC, JT, MR, GW, FF, RN, LC, AS, BF, JA, LO, AC performed investigation. MC and MJM wrote the original draft of the manuscript. RN, JA, BF, and FF reviewed and edited the manuscript. BC, DS, RKA, and MJM acquired funding. BC, DS, RKA, and MJM provided resources. MC performed visualization. MJM supervised the study.

## Conflict of interest

The authors have declared that no conflict of interest exists.

## Funding support

This work is the result of NIH funding, in whole or in part, and is subject to the NIH Public Access Policy. Through acceptance of this federal funding, the NIH has been given a right to make the work publicly available in PubMed Central.

- NIH grant T32GM008208 (awarded to University of Pittsburgh Medical Scientist Training Program, which supported MC).
- NIH grant 1F30HL170777 (to MC).
- Partially supported by UPMC Children’s Hospital of Pittsburgh Research Advisory Committee Award (to MC).

## Supplementary Material

Supplemental data

Supporting data values

## Figures and Tables

**Table 1:**
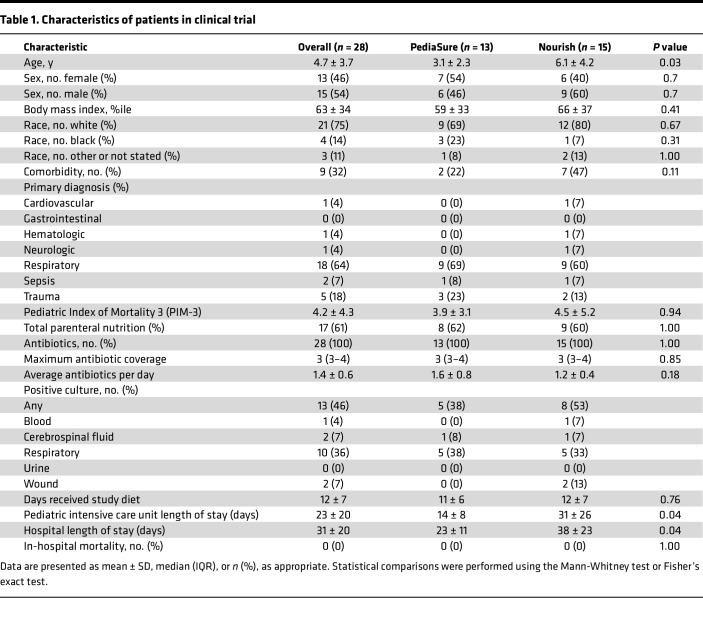
Characteristics of patients in clinical trial

## Version 1 04/09/2026

In-Press Preview

## Version 2 05/08/2026

Electronic publication
