# Supplementary material for: Plant-based enteral nutrition outperforms ultra-processed formulas in mitigating consequences of antibiotic-induced dysbiosis
Source: JCI Insight. 2026 Apr 9;11(9):e199827. doi: 10.1172/jci.insight.199827 (PMC13232488; doi:10.1172/jci.insight.199827)
Supplement: Supplemental data [file jciinsight-11-199827-s243.pdf]

Treatment	Diet	Day	Chao1 Mean	SEM	N
ABX	PBEN	0	76.29	5.59	5
ABX	PBEN	8	14.4	5.61	5
ABX	PBEN	11	5.2	0.93	5
ABX	PBEN	14	33.37	7.14	5
ABX	AEN	0	79.43	7.03	5
ABX	AEN	8	18.7	5.36	5
ABX	AEN	11	2.38	0.94	4
ABX	AEN	14	4.9	0.56	5
ABX Naive	PBEN	0	77.27	12.96	5
ABX Naive	PBEN	8	86.81	11.25	5
ABX Naive	PBEN	11	107.91	0.85	5
ABX Naive	PBEN	14	125.35	20.92	5
ABX Naive	AEN	0	64.18	8.42	5
ABX Naive	AEN	8	84.99	10.27	5
ABX Naive	AEN	11	39.65	8.39	5
ABX Naive	AEN	14	53.59	9.95	5

Table S1. Alpha diversity summary statistics (Chao1 index). Mean ± SEM for each treatment group, diet, and timepoint.

Treatment	Day	Group 1	Group 2	Mean 1	Mean 2	p-value	Sig
ABX	0	PBEN (n=5)	AEN (n=5)	76.29	79.43	0.8413	Ns
ABX	8	PBEN (n=5)	AEN (n=5)	14.4	18.7	0.6752	Ns
ABX	11	PBEN (n=5)	AEN (n=4)	5.2	2.38	0.0851	Ns
ABX	14	PBEN (n=5)	AEN (n=5)	33.37	4.9	0.0112	*
ABX Naive	0	PBEN (n=5)	AEN (n=5)	77.27	64.18	0.5476	Ns
ABX Naive	8	PBEN (n=5)	AEN (n=5)	86.81	84.99	0.9166	Ns
ABX Naive	11	PBEN (n=5)	AEN (n=5)	107.91	39.65	0.0079	*
ABX Naive	14	PBEN (n=5)	AEN (n=5)	125.35	53.59	0.0079	*

Table S2. Statistical comparisons of alpha diversity (Chao1 index). Mann-Whitney U test was used for independent comparisons between treatment groups.

	Df	Sum of Sqs	R^2	F	Pr(>F)
Days	3	4.73732	0.1469923	4.891089	0.001
Diet	1	1.196862	0.0371369	3.707133	0.001
Antibiotics	1	2.725843	0.0845791	8.442966	0.001
Residual	73	23.568324	0.7312917	NA	NA
Total	78	32.22835	1	NA	NA

Table S3. PERMANOVA analysis of weighted UniFrac distances.

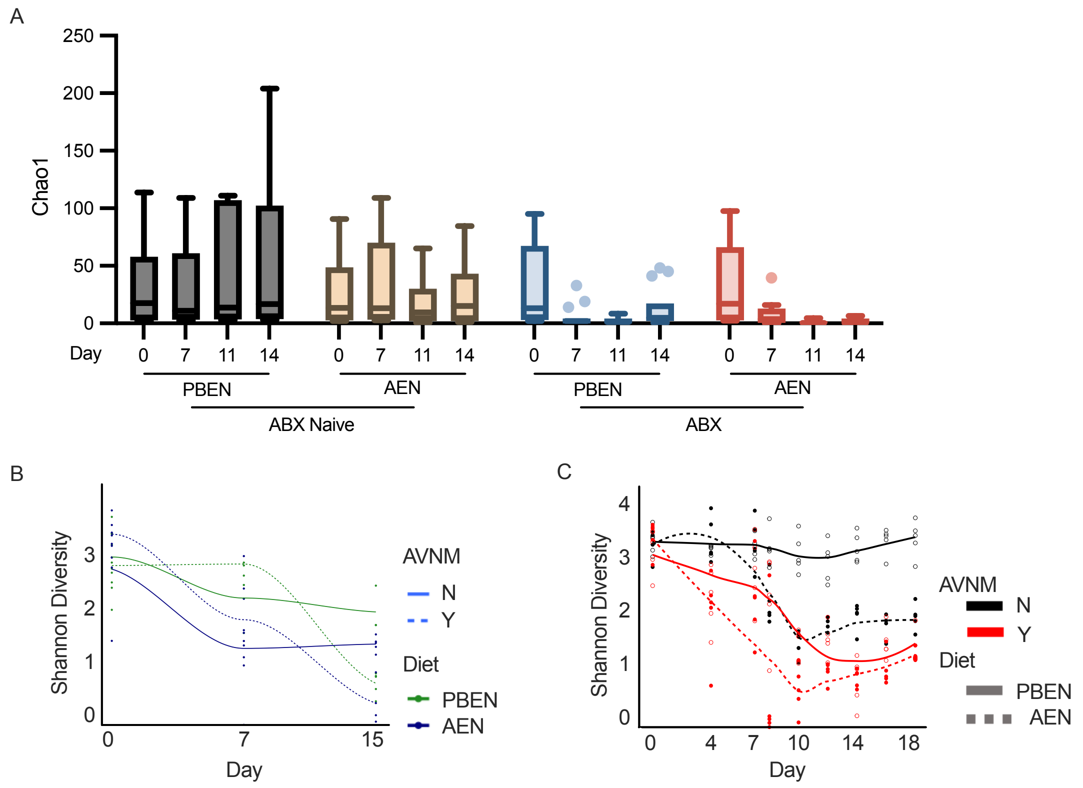

Figure S1. Alpha diversity across different experimental designs. A. Chao1 index in ABX Naive and ABX-treated mice receiving PBEN or AEN. Samples collected at baseline (Day 0), after antibiotic treatment (Day 7), and during recovery (Days 11 and 14, n=4-5 mice per group) shown as Tukey box-and-whisker plot. B. Shannon diversity index in mice that received AEN during the initial week of antibiotics (Days 0-7), then were randomized to PBEN or AEN during recovery (Days 7-15, n=4 mice per diet group). C. Shannon diversity index in mice randomized to PBEN or AEN during both antibiotic treatment (Days 0-7) and recovery (Days 7-18). Mice received AVNM in their respective diets, then continued the same diet without antibiotics for an additional ten days (n=5 mice/ diet group). Data presented as box-and-whisker plots (A) or mean with individual data points and connecting lines (B, C).

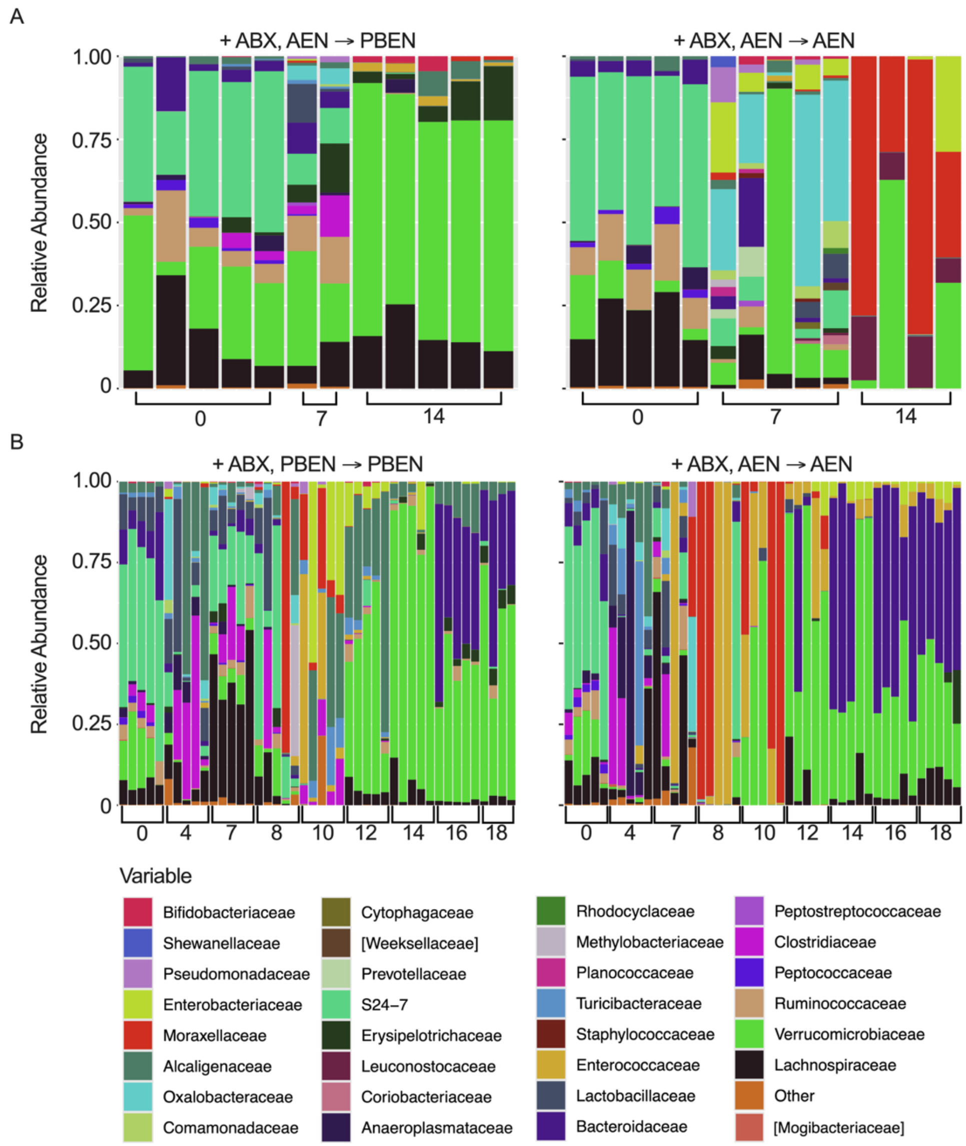

Figure S2. Diet shapes taxonomic composition following ABX exposure in independent experimental cohorts. Relative abundance of dominant bacterial families in fecal samples. **A.** Mice received AEN during initial antibiotic treatment (Days 0-7), then were randomized to PBEN (left) or AEN (right) during recovery (Days 7-14). **B.** Mice were randomized to PBEN or AEN during both antibiotic treatment (Days 0-7) and recovery (Days 7-18).Each column represents an individual mouse, n = 2-5 mice per group. Data correspond to alpha diversity in Figure S1B-C.

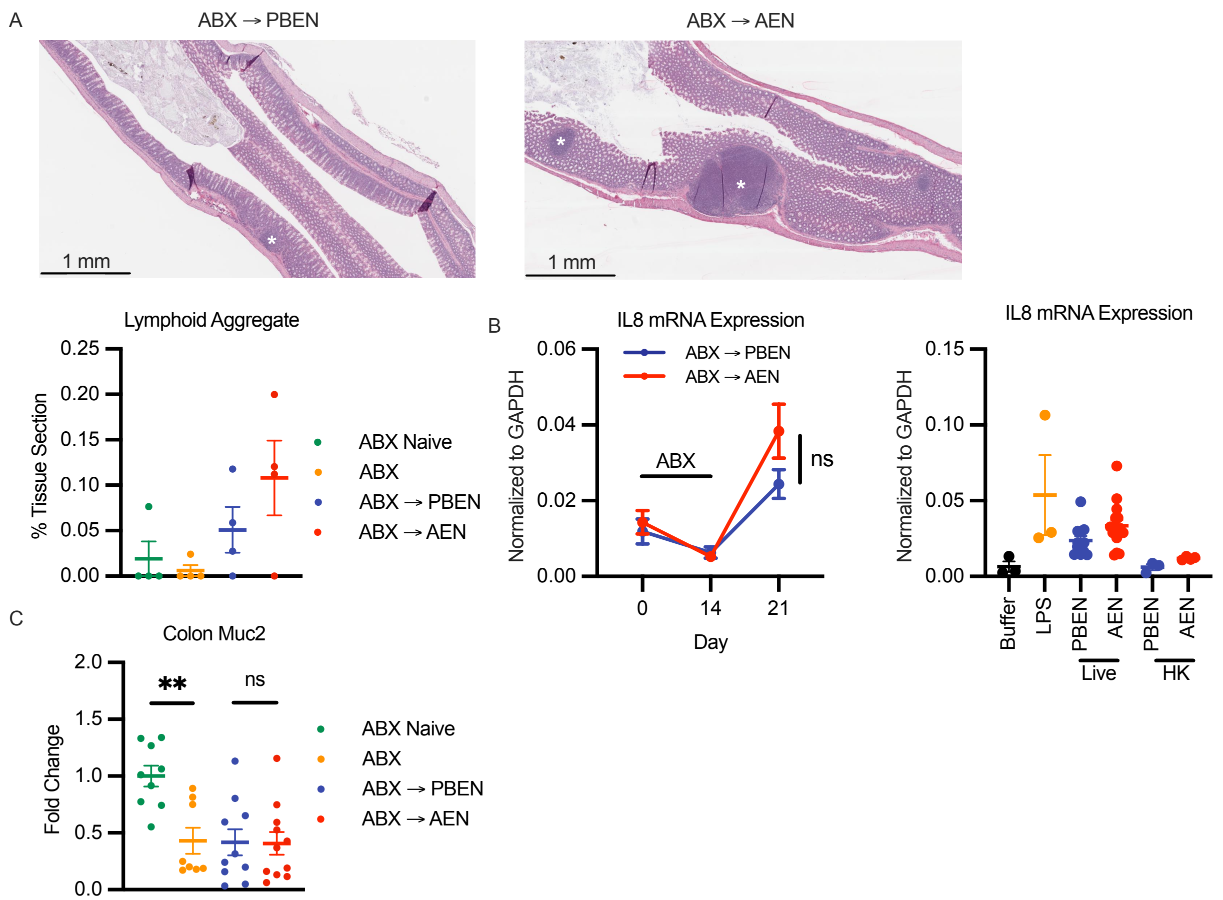

Figure S3. Antibiotic and diet-induced changes in colonic morphology and mucin gene expression. A. Representative H&E-stained colonic tissue sections from mice receiving PBEN or AEN after two weeks of antibiotic treatment. Asterisks mark lymphoid aggregates. Quantification shows the proportion of tissue occupied by lymphoid aggregates standardized to overall tissue length (4 sections per group). **B.** IL-8 mRNA expression in HT29 colonic epithelial cells co-cultured with fecal samples collected throughout antibiotic and diet treatments. Cells were exposed to either live or heat-killed bacteria derived from fecal samples. **C.** Colonic mucin-2 (MUC2) mRNA expression measured by quantitative RT-PCR (n=8-11 per group). Data represent mean ± SEM with individual data points shown. Statistical comparisons between PBEN and AEN groups were performed using Student's t-test. Data compiled from at least two independent experiments. ns, not significant; *P < .05, **P < .01, ***P < .001, ****P < .0001.

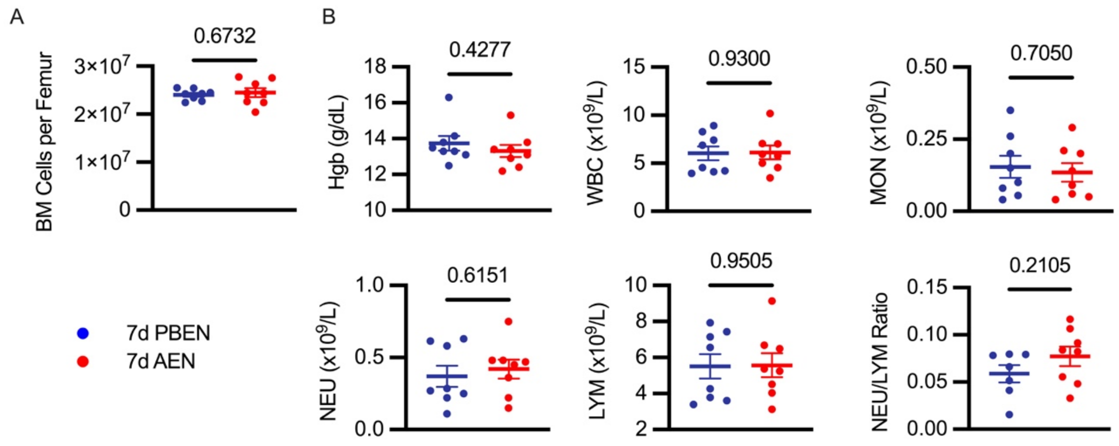

Figure S4. Bone marrow cellularity and peripheral blood profiles in the absence of antibiotic treatment. A. Bone marrow cellularity (cells per femur) in mice fed PBEN or AEN without antibiotic exposure. B. Complete blood count (CBC) profiles in ABX naive mice receiving PBEN or AEN, including hemoglobin (Hgb), total white blood cell count (WBC), monocyte count (MON), neutrophil count (NEU), lymphocyte count (LYM), and neutrophil-to-lymphocyte ratio (NLR). Data represent mean ± SEM with individual data points shown. Statistical comparisons between PBEN and AEN groups were performed using Student's t-test. n=8/ group. ns, not significant; *P < .05, **P < .01, ***P < .001, ****P < .0001.

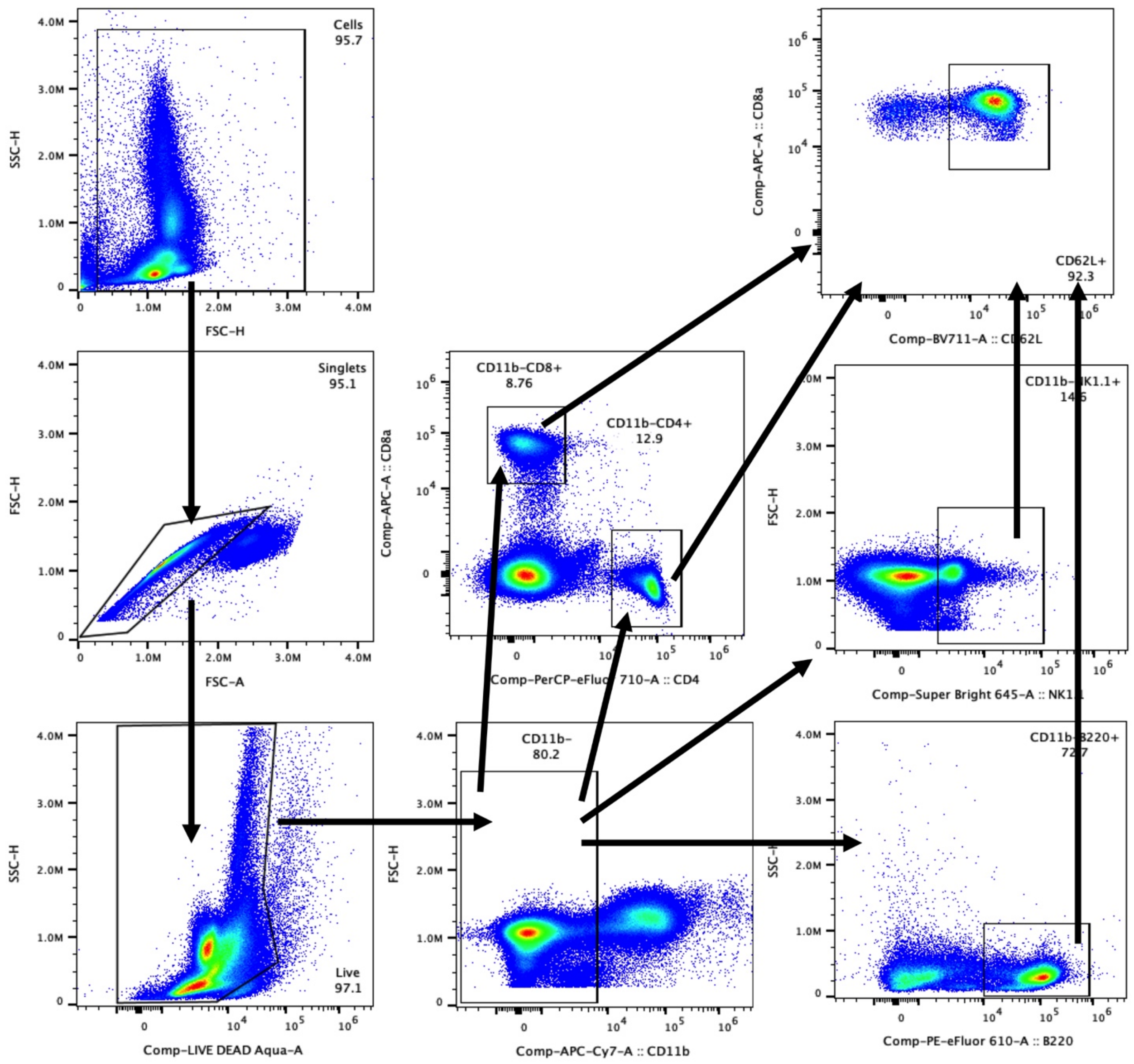

Figure S5. Flow cytometry gating strategy for bone marrow and peripheral blood analysis. Debris was excluded using side scatter (SSC) and forward scatter (FSC), followed by live cell gating with Live/Dead Aqua. CD11b$^+$ cells were gated to quantify myeloid populations. CD11b$^-$ nucleated cells were analyzed for T lymphocytes (CD4$^+$, CD8$^+$), B cells (B220$^+$), and NK cells (NK1.1$^+$). In peripheral blood, each lymphocyte population was further analyzed for CD62L expression to identify naïve versus activated cells.

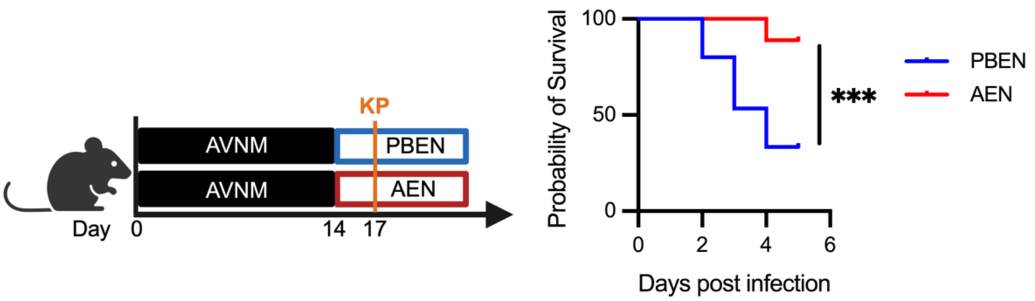

Figure S6. PBEN does not protect against Klebsiella pneumoniae-induced pneumonia following antibiotic treatment. Mice received antibiotics for two weeks followed by three days of recovery on PBEN or AEN. Mice were then infected intratracheally with *Klebsiella pneumoniae* strain KP396 and maintained on assigned diets and monitored for survival. Data represent Kaplan-Meier survival analysis. Statistical comparison performed using log-rank (Mantel-Cox) test, ***P < .001, n=16 mice/ diet.

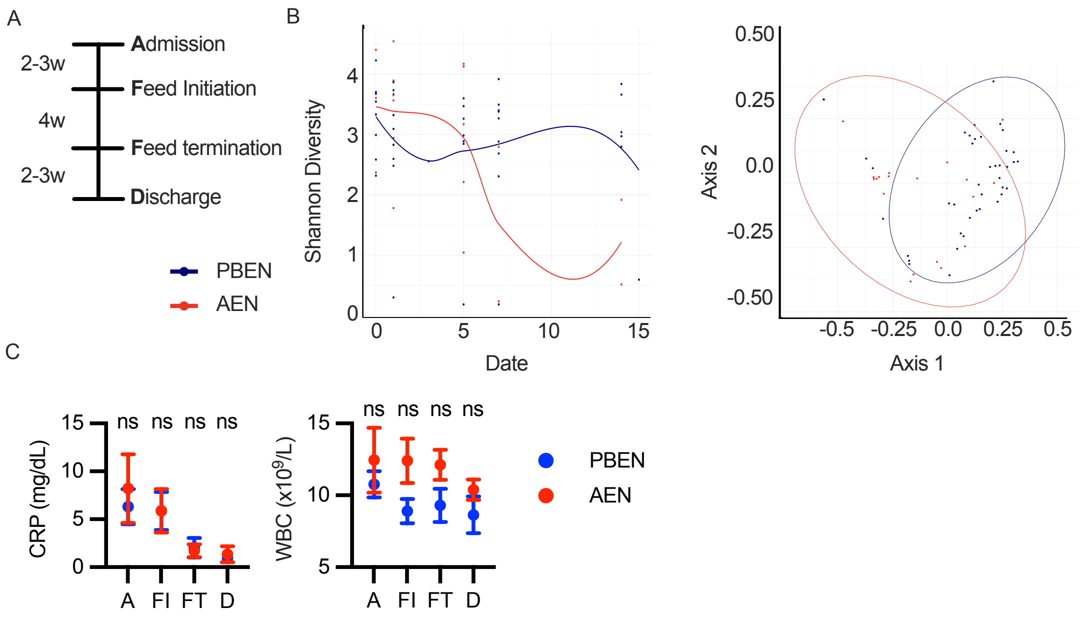

Figure S7. Dietary intervention with pediatric PBEN promotes microbiome recovery and improved peripheral blood profile in critically ill children. A. Schematic of intervention timeline. Stool/ cecal swabs and blood were collected from patients at time of admission, feed initiation with either Nourish (PBEN) or Pediasure (AEN). Measurements of B. Shannon diversity plot and unifrac analysis. B. C-reactive protein (CRP) and white blood cells (WBC). Ddata represent mean ± standard error of the mean (SEM). Significance determined by Student t test between PBEN versus AEN randomized patients.

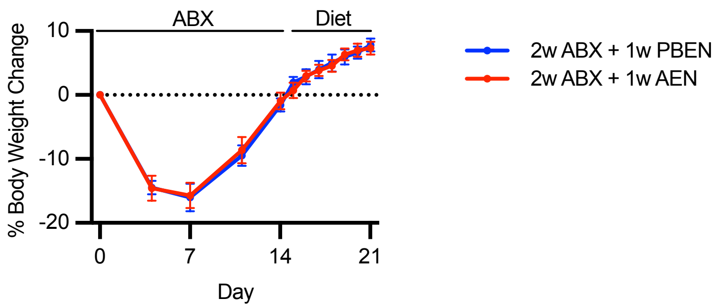

Figure S8. Body weights throughout experimental interventions. Body weights were monitored throughout antibiotic treatment and dietary intervention to trend weight loss/gain trajectories. Data represent mean ± SEM (n=6/ diet).

	Used in animal studies			Used in clinical trial	
	Liquid Hope (undiluted, per 12 fl oz)	Liquid Hope (diluted, per 8 fl oz)	Vital (per 8 fl oz)	Nourish (per 12 fl oz)	Pediasure (per 8 fl oz)
Calories	400	213	237	400	240
Protein (g)	14	7.5	9.5	14	7
Fat (g)	17	9.1	9	17	9
Carbohydrate (g)	50	26.7	30.7	50	33
Dietary Fiber (g)	7	3.7	1	7	<1
Total Sugars (g)	8	4.3	6	8	7
Added Sugars (g)	0	0	6	0	7
Vitamins & Minerals					
Vitamin D (mcg)	5	2.7	4.3	5	6
Calcium (mg)	340	181.3	205	340	330
Iron (mg)	4.2	2.2	3.0	4.2	2.7
Potassium (mg)	770	410.7	350 mg	770	470

Table S4. Nutritional profiles of plant-based enteral nutrition (PBEN) and artificial enteral nutrition (AEN) formulas used in murine studies and the human clinical trial. In animal studies, Liquid Hope (PBEN) was diluted 4:5 with water for caloric equivalence to Vital (AEN). Undiluted

and diluted and standardized per 8 fl oz shown for direct comparison. In the clinical trial, Nourish (PBEN) was diluted 4:1 with sterile water to prevent feeding tube obstruction.

PBEN formulas (Liquid Hope and Nourish) differ substantially from AEN formulas (Vital and Pediasure) not only in macronutrient composition but also in the source and type of ingredients used. A detailed list of ingredients for each formula is provided below to highlight these differences.

Liquid Hope and Nourish: Filtered water, organic garbanzo beans, organic green peas, organic whole grain brown rice, organic carrots, organic extra virgin olive oil, organize sprouted quinoa, organic pea protein, vitamin blend, organic sweet potato, organic broccoli, organic kale, organic acerola powder, organic flax oil, organic turmeric, organic ginger.
Vital: Water, Corn Maltodextrin, Whey Protein Hydrolysate, Structured Lipid (Interesterified Canola Oil & Medium Chain Triglycerides), Sugar, Hydrolyzed Sodium Caseinate. Less than 1% of: Canola Oil, Medium Chain Triglycerides, Short-chain Fructooligosaccharides, Natural & Artificial Flavor, etc

Pediasure: Water, Corn Maltodextrin, Blend of Vegetable Oils (Soy, High Oleic Safflower), Sugar, Milk Protein Concentrate, Soy Protein Isolate. Less than 0.5% of: Vitamins & Minerals, Natural & Artificial Flavors, Cellulose Gel, Tuna Oil, Cellulose Gum, Monoglycerides, Soy Lecithin, Carrageenan, Potassium Hydroxide, Inositol, Salt, Taurine, Stevia Leaf Extract, L-Carnitine, and Lutein, etc.

Supplementary Methods

Coculture of stool with HT29 cell line

Stool samples were homogenized in PBS, and bacteria were isolated for coculture by homogenizing in PBS and filtering. The bacterial pellet was resuspended in PBS containing 25% glycerol for storage. Quantification of bacterial concentration was achieved by measuring the optical density (OD) of the suspension at 600 nm. To obtain a standardized inoculum for coculture experiments, the bacterial concentration was adjusted to an OD of 0.05. For heat-inactivated controls, an aliquot of the bacterial suspension was incubated at 70°C for 30 minutes. For coculture, HT29 cells were seeded at a density of 300,000 cells per well in a 12-well plate the night before coculture and incubated at 37°C overnight in RPMI media supplemented with 10% FBS. The next day, 60 μL of the standardized bacterial homogenate was added to the HT29 cell wells and then incubated for 6 hours. RNA was collected and processed for qPCR analysis.

Klebsiella pneumoniae intratracheal infection

Mice received antibiotics for two weeks, followed by three days recovery on PBEN or AEN. Mice were infected with a hypervirulent mucoid K1 serotype clinical isolate of *Klebsiella pneumoniae* (KP396; gifted from Campfield Lab, UPMC Children's Hospital, Pittsburgh, PA, USA) and maintained on assigned diets for seven days (1). KP396 was grown overnight at 37°C with shaking in tryptic soy broth, diluted to 8,000 CFU/L, and administered intratracheally (400 CFU/mouse).

Key resources table

Antibodies

Antibody	Source	Identifier
Rat monoclonal anti-mouse CD11b	Fisher Scientific	Cat no. 557657<br>RRID:AB_396772
Rat monoclonal anti-mouse Gr1	Invitrogen	Cat no. 25-5931-82<br>RRID: AB_469663
Rat monoclonal anti-mouse CD62L	Biolegend	Cat no. 104445<br>RRID:AB_2564215
Rat monoclonal anti-mouse CD4	Invitrogen	Cat no. 46-0041-82

		RRID:AB_11150050
Rat monoclonal anti-mouse CD8a	Invitrogen	Cat no. 17-0081-82<br>RRID:AB_469335
Rat monoclonal anti-mouse CD45R (B220)	Invitrogen	Cat no. 61-0452-82<br>RRID:AB_2574558
Mouse monoclonal anti-mouse NK1.1	Invitrogen	Cat no. 64-5941-82<br>RRID:AB_2662737
Rat monoclonal anti-mouse CD16/32	Biolegend	Cat no. 101301<br>RRID:AB_312800

Bacterial and virus strains

Bacteria/Virus	Source
Vancomycin Resistant *Enterococcus faecium* strain 34-10-S	Van Tyne Lab, University of Pittsburgh, Pittsburgh, PA, USA
Klebsiella Pneumoniea strain 396 (1)	Campfield Lab, UPMC Children's Hospital Pittsburgh, Pittsburgh, PA, USA

Chemicals

Chemical	Source	Identifier
Ampicillin	Sigma Aldrich	Cat no. A0166
Vancomycin	Thermo Fisher Scientific	Cat no. J62790.06
Neomycin	Sigma Aldrich	Cat no. N1876
Metronidazole	Sigma Aldrich	Cat no. M3761
RPMI 1640 Medium	Life Technologies	Cat no. 11875085
FBS	Corning	Cat no. MT35011CV
HBSS	Life Technologies	Cat no.14170112
High capacity RNA-to-cDNA kit	ThermoFisher Scientific	Cat no. 4387406
Power SYBR Green PCR Master Mix	ThermoFisher Scientific	Cat no. 4367659
Lipopolysaccharides (LPS) from Escherichia coli O111:B4	Millipore Sigma	Cat no. L2630
Dextran sulfate sodium	ThermoFisher Scientific	Cat no. AAJ6360622
Protease and phosphatase inhibitor	Thermo Fisher	Cat no. 78441
BHI medium	BD Biosciences	Cat no. 237500

Commercial assays

Assay	Source	Identifier
Lipocalin ELISA	R&D Systems	Cat no. DY1857
ProcartaPlex Mouse Cytokine and Chemokine Panel 1A	ThermoFisher Scientific	Cat no. EPX360-26092-901
Reg3g ELISA	MyBioSource	Cat no. MBS2705572
BCA Assay	ThermoFisher Scientific	Cat no. 23227
TNFa ELISA	R&D Systems	Cat no. DY410
IL-10 ELISA	R&D Systems	Cat no. DY417

Cell lines and organisms

Cell line/ organism	Source	Identifier
HT29	ATCC	Cat no. HTB-38<br>RRID:CVCL_0320
C57BL/6 Mice	Jackson Laboratories	RRID: IMSR_JAX:000664

Oligonucleotides

Target	Forward	Reverse
Mouse Reg3g	CGTGCCTATGGCTCCTATTGCT	TTCAGCGCCACTGAGCACAGAC
Mouse MUC2	GCCCACCTCACAAGCAGTAT	GTCATAGCCAGGGGCAAACT
Human IL8	GAGAGTGATTGAGAGTGGACCAC	CACAACCCTCTGCACCCAGTTT
Mouse KP396 (ZKIR)	CTAAAACCGCCATGTCCGATTTAA	TTCCGAAAATGAGACACTTCAGA

Other resources

Cell line/ organism	Source	Identifier
Blood agar plates	ThermoFisher Scientific	Cat no. R01202
AOPI staining solution	Revvity	Cat no. CS2-0106
Red cell lysis buffer	Sigma Aldrich	Cat no. R7757
Pierce BCA Protein Assay Kit	ThermoFisher Scientific	Cat no. PI23225
Zymo Research Quick RNA MiniPrep	Zymo Research	Cat no. R1055
QIAzol Lysis Reagent	Qiagen	Cat no. 79306
BD BBL Enterococcosel Agar	ThermoFisher Scientific	Cat no. B12205
Flow Cytometry Staining Buffer	ThermoFisher Scientific	Cat no. 00-4222-26
BD BBL CultureSwab EZ	Fisher Scientific	Cat no. B220144
DNeasy 96 PowerSoil Pro QIAcube HT Kit	Qiagen	Cat no. 47021

Software and Algorithms

Software/ Algorithm	Source
FlowJo	https://www.flowjo.com
GraphPad Prism 9	https://www.graphpad.com
Quantitative Insights into Microbial Ecology 2 (QIIME2)	https://github.com/qiime 2/
Greengenes taxonomic database (version 13.5)	https://github.com/knight lab-analyses/greengenes2<br><br>https://greengenes2.ucsd.edu
FastTree	http://www.microbesonli ne.org/fasttree
R Phyloseq library	https://github.com/joey7 11/phyloseq/issues<br><br>https://www.bioconducto r.org/packages/release/b ioc/html/phyloseq.html
VEGAN	Dixon (2)
Analysis of Composition of Microbiomes 2 (ANCOM2)	https://github.com/Frede rickHuangLin/ANCOM-BC

Cutadapt	https://cutadapt.readthe docs.io/en/stable/
Trimmomatic	http://www.usadellab.org /cms/index.php?page=tri mmomatic
Burrows Wheeler Alignment (BWA)	https://github.com/Lilu-guo/Effaln
Kraken2	https://github.com/Derric kWood/kraken2
Samtools	http://www.cazy.org
DIAMOND	https://github.com/bbuchfink/diamond
HMMER	https://github.com/Eddy RivasLab/hmmer<br><br>http://hmmer.org

Bibliography

1. Chen K, et al. Th17 cells mediate clade specific, serotype-independent mucosal immunity. *Immunity*. 2011;35(6):997–1009.
2. Dixon P. VEGAN, a package of R functions for community ecology. *Journal of Vegetation Science*. 2003;14(6):927–930.
